# Supplementary material for: TMBIM6/BI-1 contributes to cancer progression through assembly with mTORC2 and AKT activation
Source: Nat Commun. 2020 Aug 11;11:4012. doi: 10.1038/s41467-020-17802-4 (PMC7419509; doi:10.1038/s41467-020-17802-4)
Supplement: Supplementary file 1 — Supplementary Information [file 41467_2020_17802_MOESM1_ESM.pdf]

Supplementary Information for

TMBIM6/BI-1 contributes to cancer progression through assembly with mTORC2 and AKT activation

Hyun-Kyoung Kim *et al*.

Supplementary Fig. 1

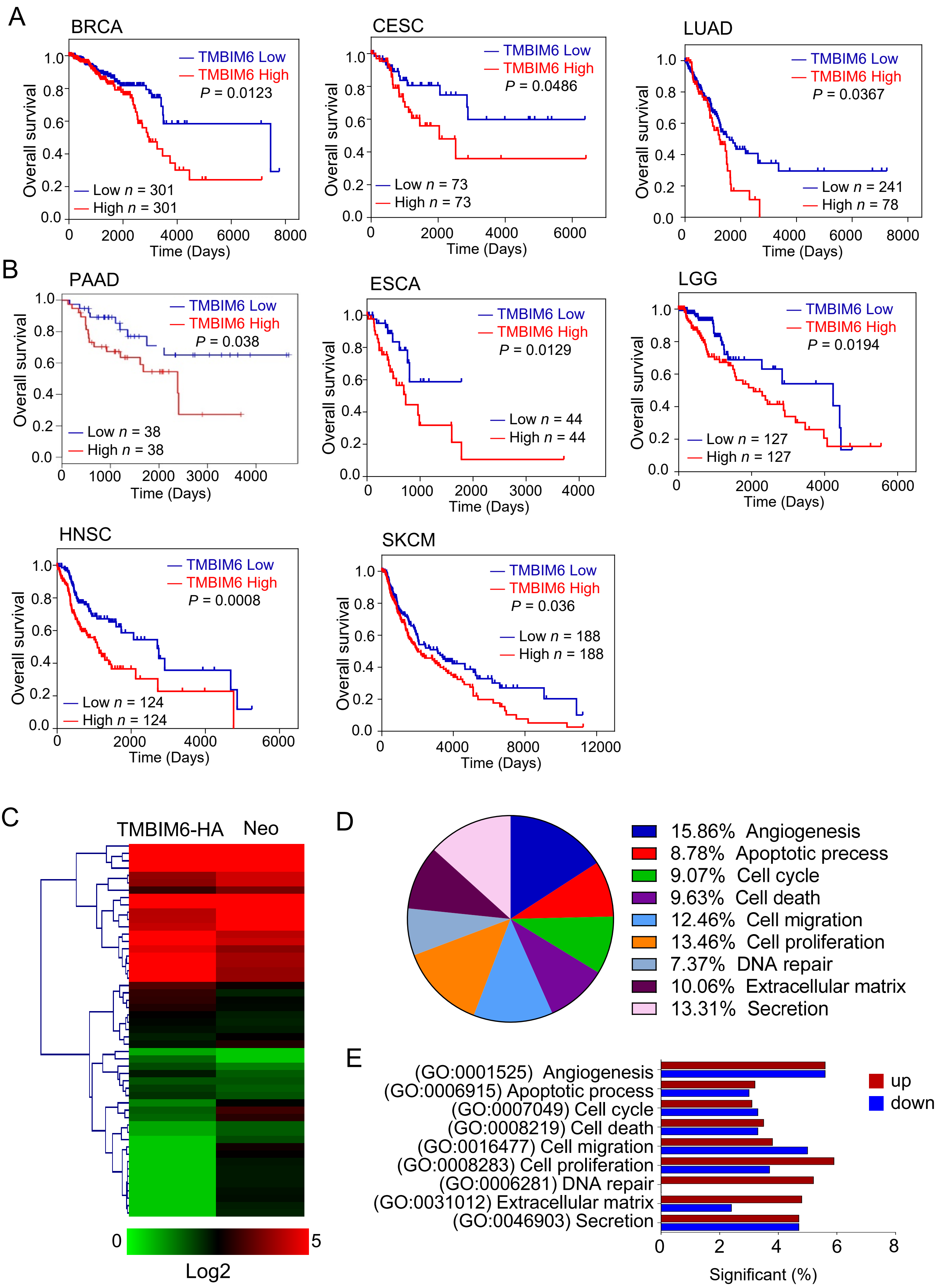

Supplementary Fig. 1 (A-B) Kaplan-Meier curves showing the overall survival analysis in patients with high and low expression of TMBIM6 using OncoLnc from TCGA database and GEPIA2. P value with log-rank analysis. BRCA; breast invasive carcinoma, CESC; cervical squamous cell carcinoma and endocervical adenocarcinoma, LUAD; lung adenocarcinoma, PAAD; pancreatic adenocarcinoma, ESCA; esophageal carcinoma, SKCM; skin cutaneous melanoma, HNSC; head and neck squamous cell carcinoma, LGG; brain lower grade glioma. (C) Microarray data analysis shows heat map by hierarchical clustering of genes expressed in TMBIM6-overexpressing HT1080 cells. (D) Significant ratios in TMBIM6-overexpressing HT1080 cells determined by Gene Ontology analysis. (E) The graph indicates significant differences in downregulation and upregulation of the indicated category genes in TMBIM6-overexpressing HT1080 cells compared with Neo-control cells.

Supplementary Fig. 2

A

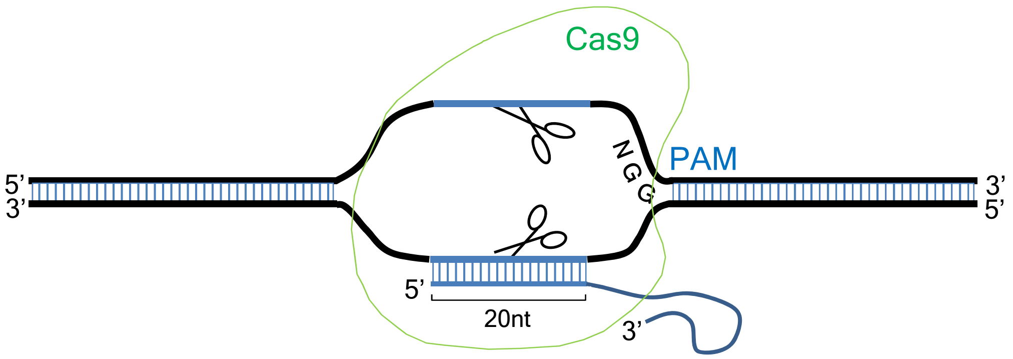

Human *TMBIM6* locus

PAM

5'-GTTTGTGGCGGCTGCAGGGGCCTATGTCCATA**TGG**TCACTCATTTCATTC-3'

3'-CAAACACCGCCGACGTCCCCGGATACAGGTATACCAGTGAGTAAAGTAAG-5'

5'-UGCAGGGGCCAUACAGGAUA-3' sgRNA

HT1080

#1	Allele 1	5'-GTTTGTGGCGGCTGCAGGGGCCTA·······ATGGTCACTCATTTCATTC-3'
	Allele 2	5'-GTTTGTGGCGGCTGCAGGGGCCTATGTC··········CTCATTTCATTC-3'
#2	Allele 1	5'-GTTTGTGGCGGCTGCAGGGGCCTATGT·CATATGGTCACTCATTTCATTC-3'
	Allele 2	5'-GTTTGTGGCGGCTGCAGG·················TCACTCATTTCATTC-3'

HeLa

#1	Allele 1	5'-GTTTGTGGCGGCTGCAGGGGCCTATGT·CATATGGTCACTCATTTCATTC-3'
	Allele 2	5'-GTTTGTGGCGGCTGCAGGGGCCTATGT··ATATGGTCACTCATTTCATTC-3'
#2	Allele 1	5'-GTTTGTGGCGGCTGCAGGGGCCTA·······ATGGTCACTCATTTCATTC-3'
	Allele 2	5'-GTTTGTGGCGGCTGCAGGGGCCTATGTC··········CTCATTTCATTC-3'

B HT1080

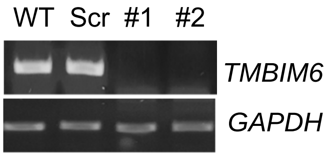

C HeLa

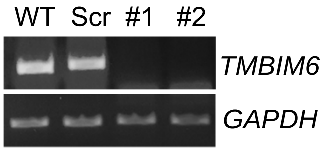

Supplementary Fig. 2 Generation of TMBIM6 KO cells by CRISPR/Cas9 genome editing. (A) Schematic illustration of genome editing via CRISPR/Cas9-mediated RNA-guided site-specific DNA cleavage. The sequence of the mutated allele in TMBIM6 harboring the insertions/deletions is shown. (B-C) *TMBIM6* mRNA levels in WT and TMBIM6-KO HT1080 (B) and HeLa cells (C), as determined by qRT-PCR. Blots represent one out of two experiments, with similar results obtained.

Supplementary Fig. 3

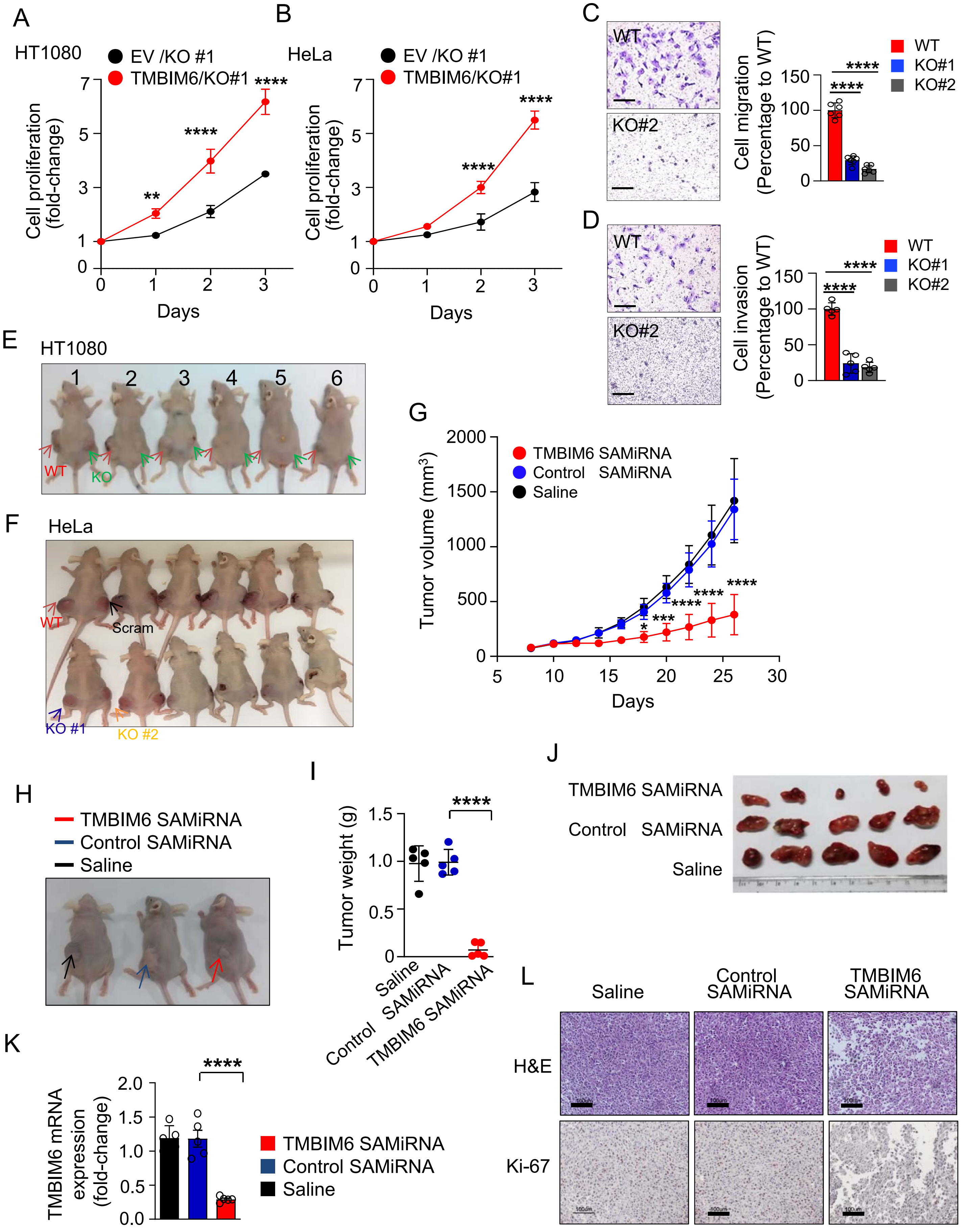

Supplementary Fig. 3 TMBIM6 regulates tumor growth. (A-B) Proliferation of rescue TMBIM6 expressing in TMBIM6 KO HT1080 (A) and HeLa (B) cells (n = 3 independent experiments). Data are presented as means ± SD. **p < 0.01, ****p < 0.0001, two-way ANOVA followed by Bonferroni's post hoc test. (C-D) Images and quantification of migrated cells (C) or invasive cells (D) represented in TMBIM6 KO and WT HT1080. Quantification data represented percentage of WT cells normalized to KO cells (n = 6 for migration, n = 5 for invasion independent experiments). Scale bars, 100 μm. Data are presented as means ± SD. ****p < 0.0001, one-way ANOVA followed by Tukey's post hoc test. (E-F) Representative images of tumors from xenograft experiments shown in Figure 2D and Figure 2H. (G-J) Tumor volume, weight, and image derived from HT1080 cells in nude mice injected via the tail vein with TMBIM6-targeted SAMiRNA, control SAMiRNA, or saline (n = 5 mice per group). Data are presented as means ± SD. *p < 0.05, ***p < 0.001, ****p < 0.0001, two-way ANOVA followed by Bonferroni's post hoc test in G and one-way ANOVA followed by Tukey's post hoc test in I. (K) mRNA levels of TMBIM6 in tumors derived from HT1080 cells following TMBIM6 siRNA treatment, as determined by qRT-PCR (n = 5 mice per group). Data are presented as means ± SD. ****p < 0.0001, one-way ANOVA followed by Tukey's post hoc test. (L) Hematoxylin and eosin staining and immunodetection of Ki-67 in tumors derived from HT1080 cells in nude mice injected via the tail vein with TMBIM6-targeted SAMiRNA, control SAMiRNA, or saline. Images represent one out of five mice xenografts, with similar results obtained.

Supplementary Fig. 4

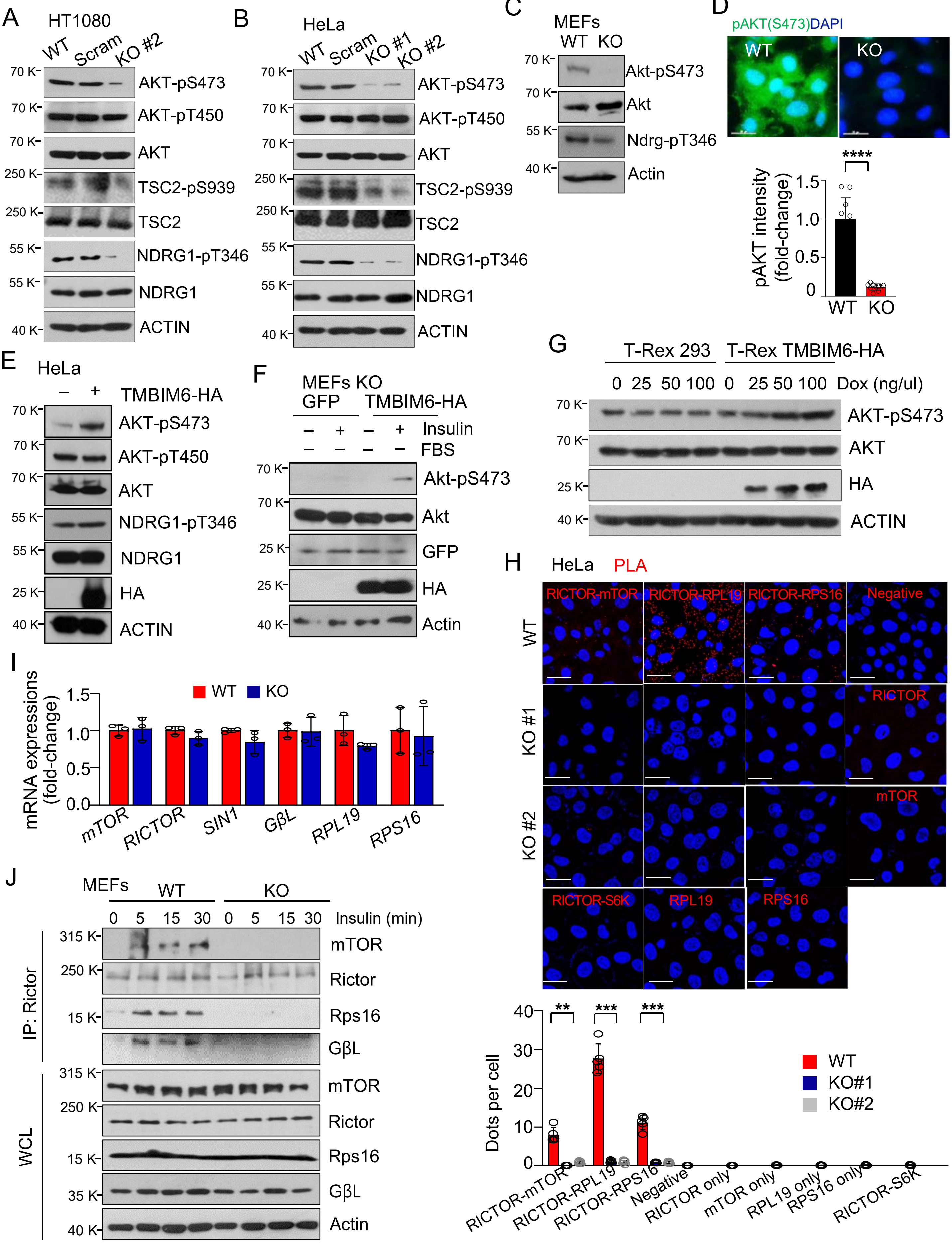

Supplementary Fig. 4 TMBIM6 regulates mTORC2 activity. (A-C) Immunoblot analysis of indicated proteins in HT1080, HeLa, and MEFs cells. (D) Immunofluorescence images of pAKT. Right, mean pAKT intensity in the KO cells normalized to WT cells (n = 10 independent experiments). Data are presented as means ± SD. ****p < 0.0001, two-tailed unpaired t-test. (E) Immunoblot analysis for the indicated proteins in HeLa cells transfected with HA-TMBIM6. (F) After serum starvation for 12 h, TMBIM6 KO MEF cells with transfection of TMBIM6-HA were stimulated with or without insulin (100 ng/ml), and immunoblotting was performed using indicated antibodies. (G) Immunoblot of the indicated proteins in TMBIM6 T-Rex 293 cells treated with various concentrations of doxycycline for 24 h. (H) PLA between indicated proteins (red dots) in TMBIM6 KO and WT HeLa cells. The ribosomal protein S6 kinase beta-1 (S6K1) as negative controls for PLA was used. Scale bar, 15 μm. Bottom, quantification of red dots (n = 5 independent experiments). Data are presented as means ± SD. **p < 0.01, ****p < 0.0001, two-way ANOVA followed by Bonferroni's post hoc test. (I) mRNA levels of indicated genes in TMBIM6 KO and WT HT1080 cells, as determined by qRT-PCR (n = 3 independent experiments). Data are presented as means ± SD. Two-way ANOVA followed by Bonferroni's post hoc test was used. (J) Immunoblot analysis of anti-RICTOR IP and WCL of TMBIM6 KO and WT MEFs. WCL, whole cell lysates.

Supplementary Fig. 5

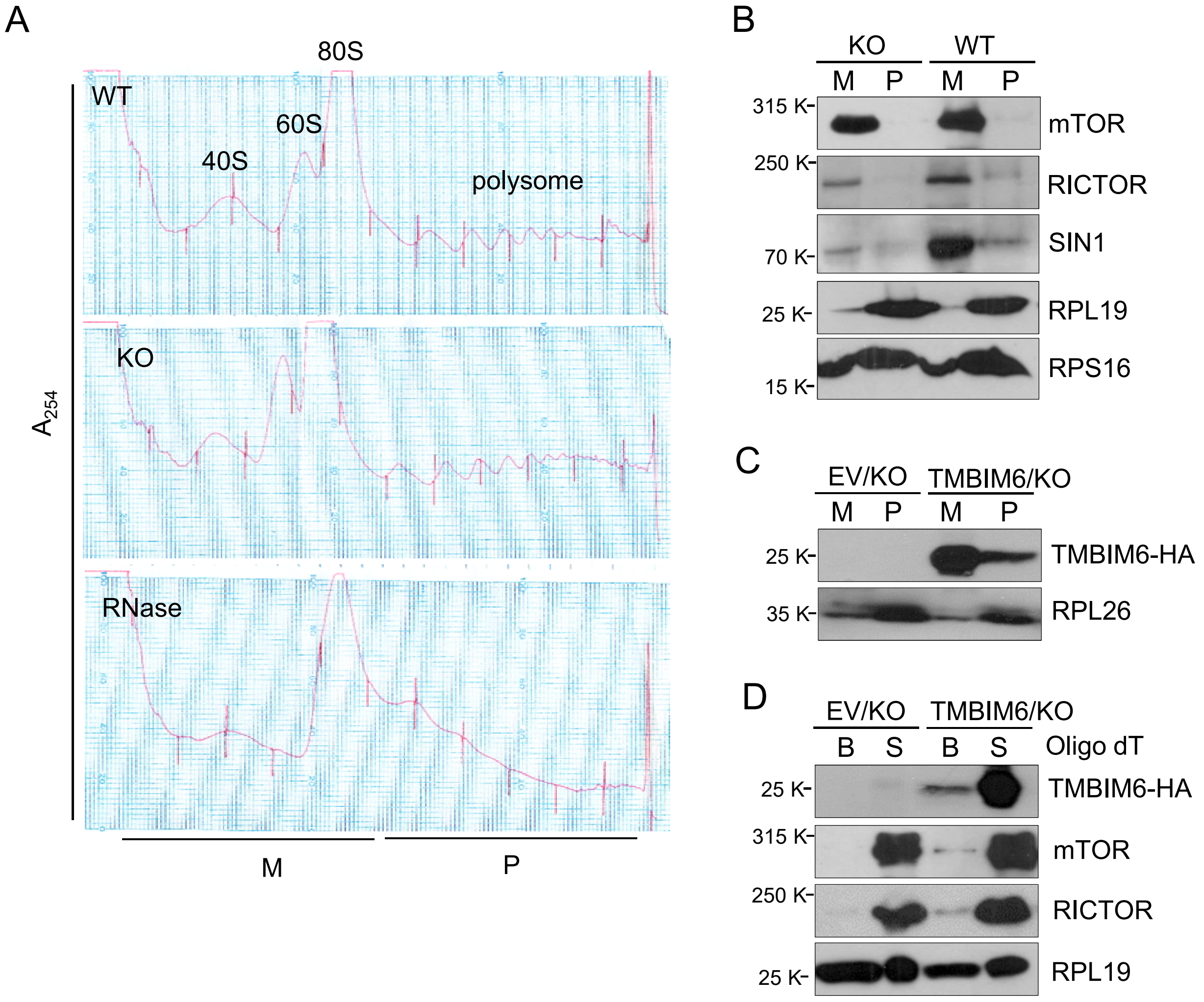

Supplementary Fig. 5 TMBIM6 regulates the association of mTORC2 and ribosome. (A) Polysome profiling was performed in TMBIM6 WT and KO HT1080 cells by sucrose gradient fractionation. The polysomal (P) and ribosomal (M) fractions are indicated. (B) Immunoblot analysis with the indicated antibodies of fractions from A. (C) Immunoblot analysis with the indicated antibodies of fractions from empty-vector and TMBIM6 rescued KO HT1080 cells. (D) Immunoblot analysis with the indicated antibodies was performed in the purified poly(A) mRNA-bound ribosomes from HT1080 cell with stably expressing TMBIM6 by oligo(dT) pull-down. The bound fraction (bound) and the supernatant are indicated. Blots represent one out of two experiments (B, C, D), with similar results obtained.

Supplementary Fig. 6

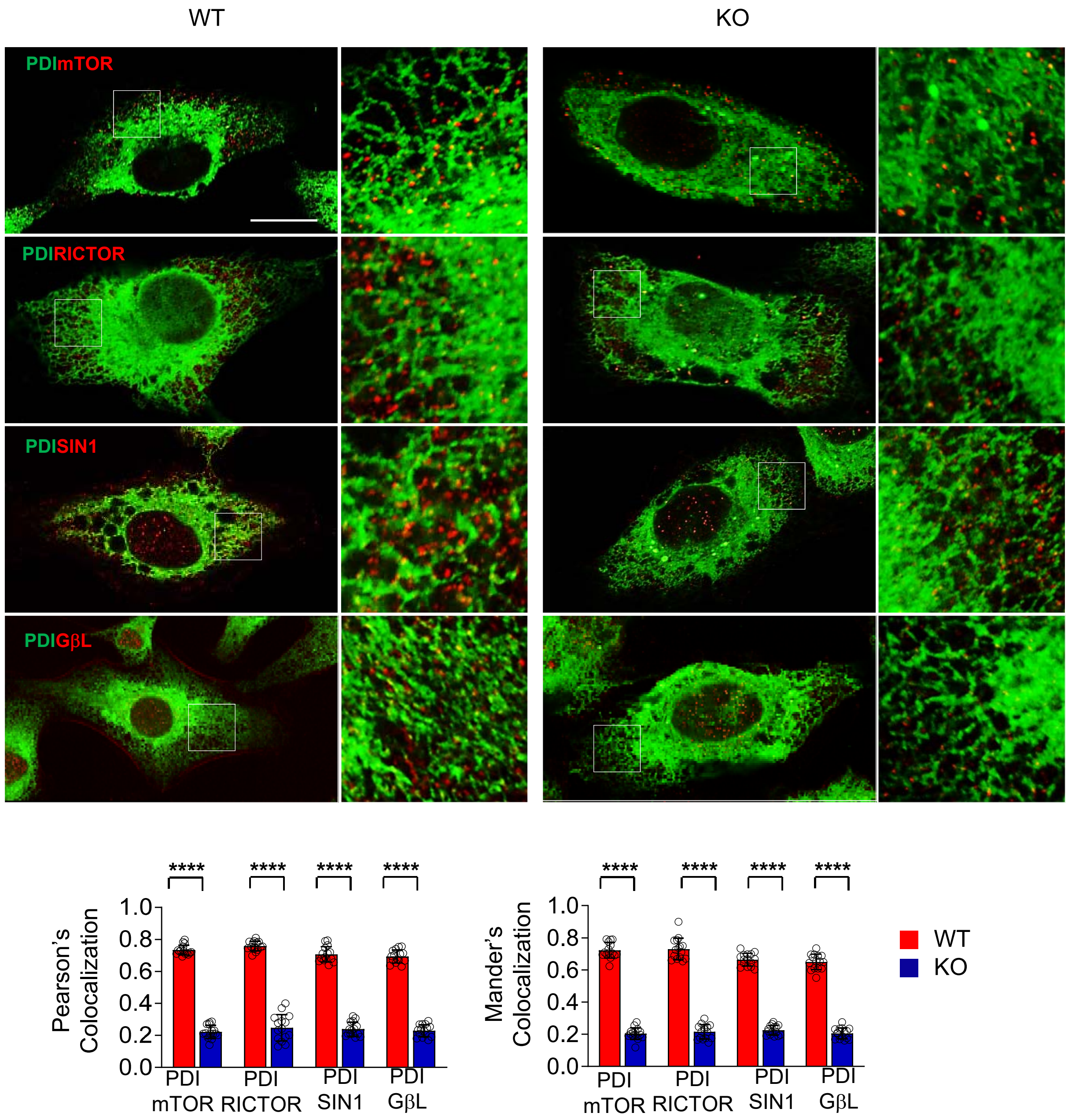

Supplementary Fig. 6 TMBIM6 regulates mTORC2 residency on the endoplasmic reticulum. Cells were stained for PDI (ER marker) and mTORC2 components by indirect immunofluorescence (upper) followed by colocalization quantification (bottom; n = 15 cells). Scale bar, 10 and 5 μm. Bottom, quantification of the dot intensity in the TMBIM6 KO cells normalized to WT cells. Data are presented as means ± SD. ****$p < 0.0001$, two-way ANOVA followed by Bonferroni's post hoc test.

Supplementary Fig. 7

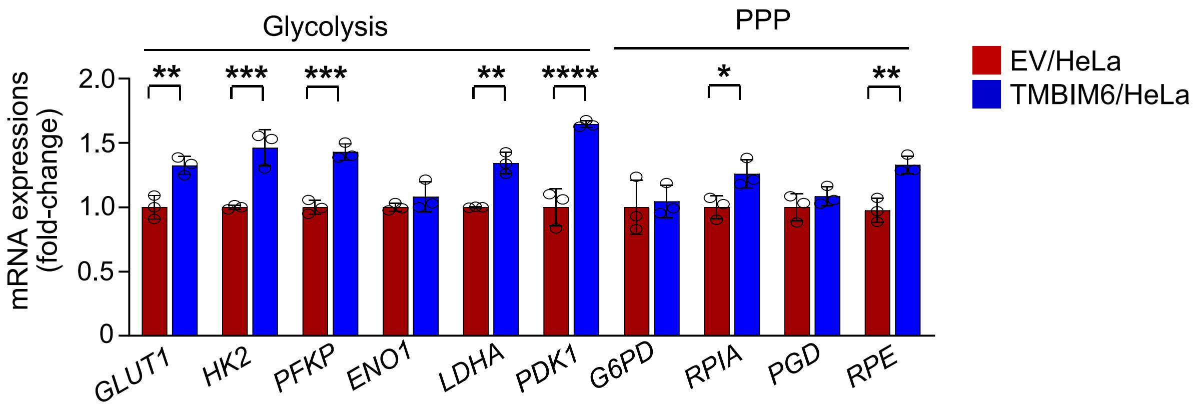

Supplementary Fig. 7 TMBIM6 regulates cell metabolism. mRNA levels of glycolysis- and PPP-related genes in TMBIM6-overexpressing HeLa cells, as determined by qRT-PCR. Quantification data represent normalized empty vector (EV) cells after normalization to the level of β-actin (n = 2 independent experiments). Data are presented as means ± SD. *$p < 0.05$, **$p < 0.01$, ***$p < 0.001$, ****$p < 0.0001$, two-way ANOVA followed by Bonferroni's post hoc test.

Supplementary Fig. 8

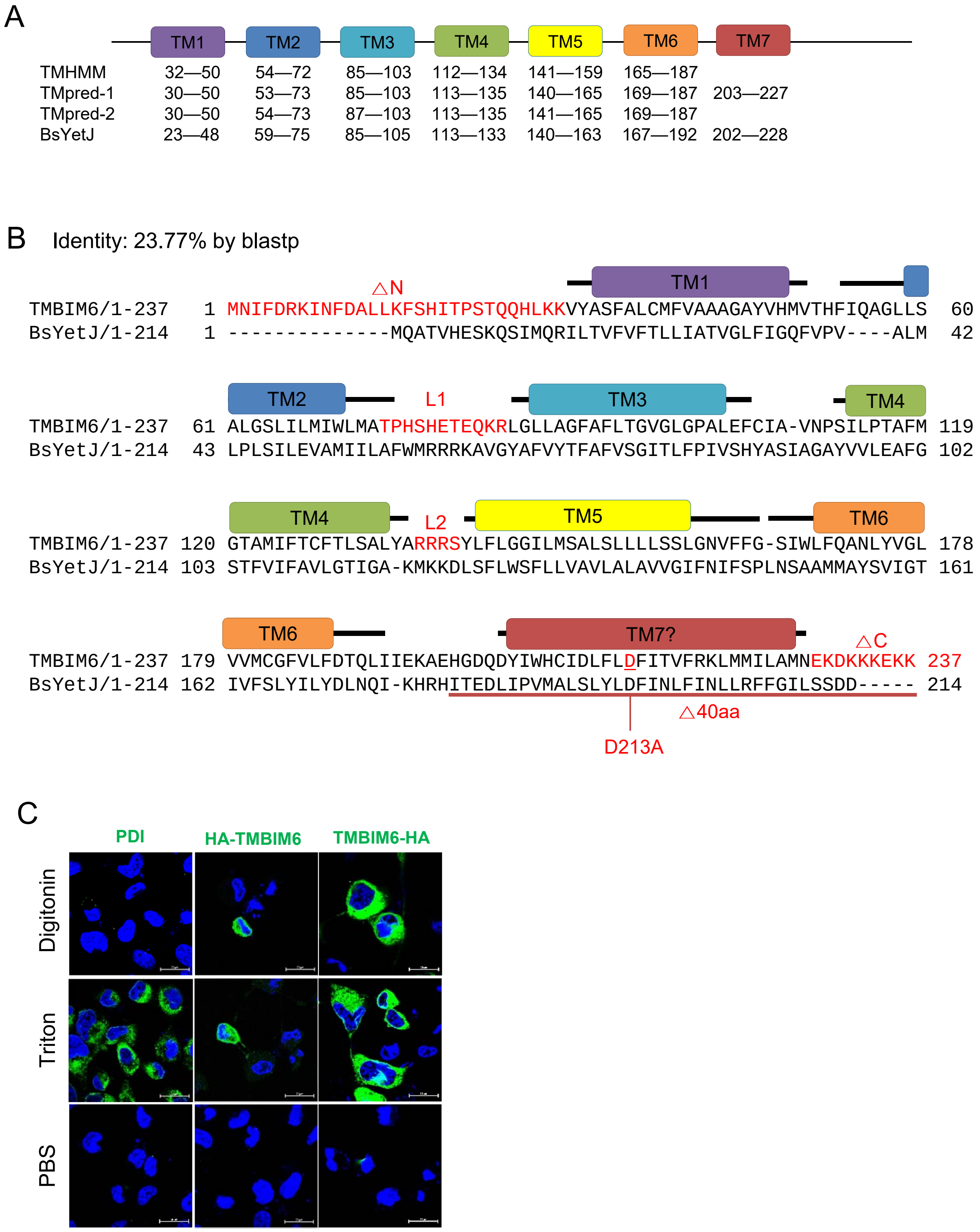

Supplementary Fig. 8 Representative TMBIM6 topology. (A) Bioinformatical prediction of the topology of TMBIM6 according to TMpred, TMHMM, and BsYetJ. The box and number indicate transmembrane domain and amino acids, respectively. (B) Alignment of amino acid sequences between TMBIM6 and BsYetJ based on previous reports. The box and line represent same and alternative prediction sequences from (A), respectively. (C) Immunofluorescence using cells overexpressing TMBIM6 tagged with the N-terminal (HA-TMBIM6) and C-terminal (TMBIM6-HA) HA tag after permeabilization by digitonin or triton X-100. Images represent one out of five experiments, with similar results obtained.

Supplementary Fig. 9

A

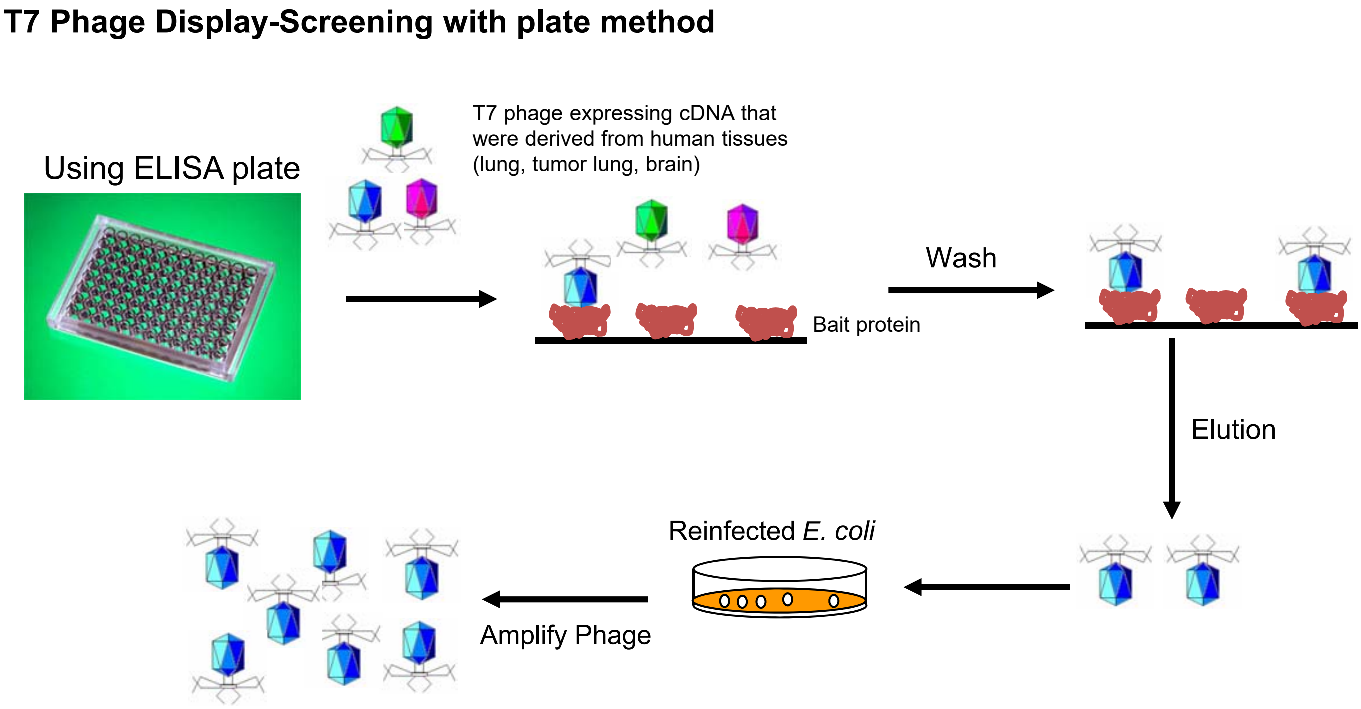

B

Sequence	Gene
SSIDRHMYHSLYLKVKGNVFKNKRILMEHIHKLKADKARKKLL ADQAEARRSKTKEARKRREERLQAKKEEIIKTLSKEEETKK	*RPL19*
SSVITSVKRWQKGQREFKRISRSIRKLQC	*sFRP-2*

Supplementary Fig. 9 Identification of TMBIM6-interacting protein. (A) Schematic illustration of protocol for T4 phage display screening by the plate method. (B) Amino acid sequence of the identified protein.

Supplementary Fig. 10

A

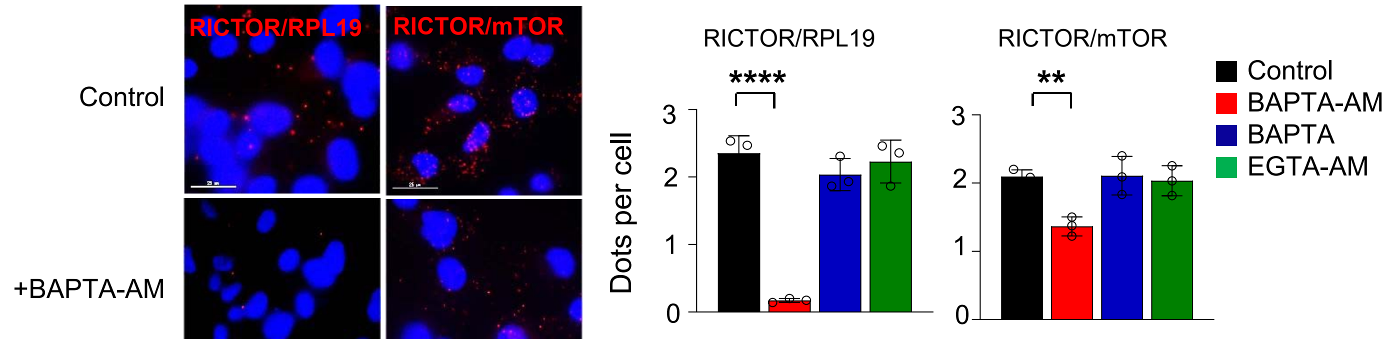

B

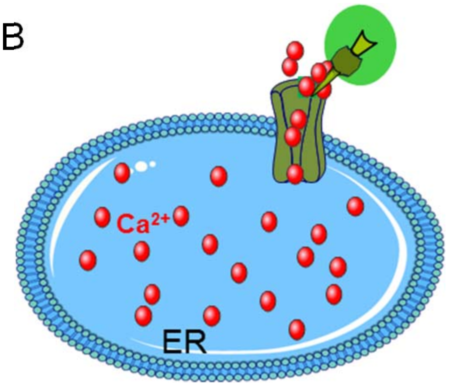

C

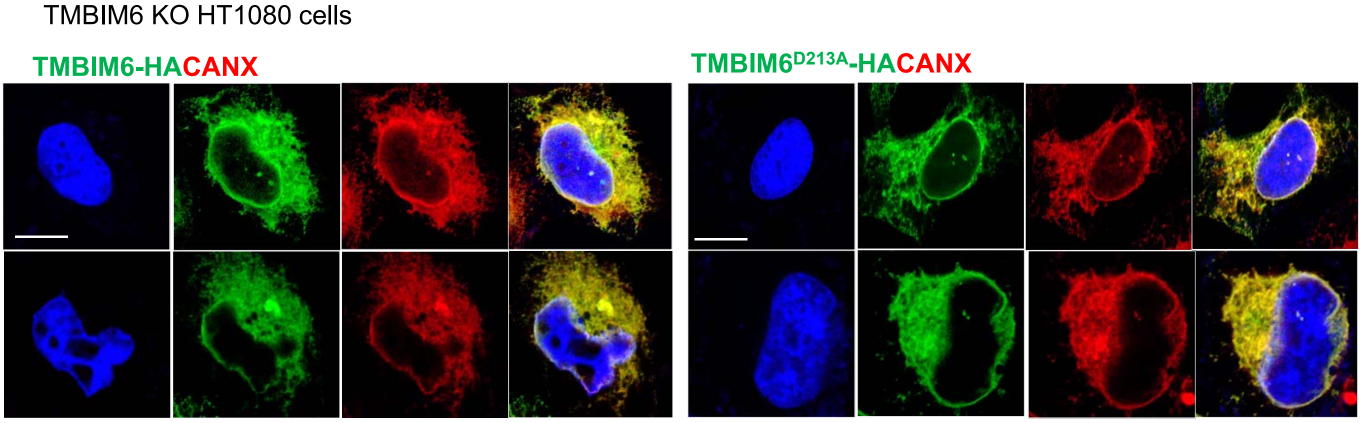

D

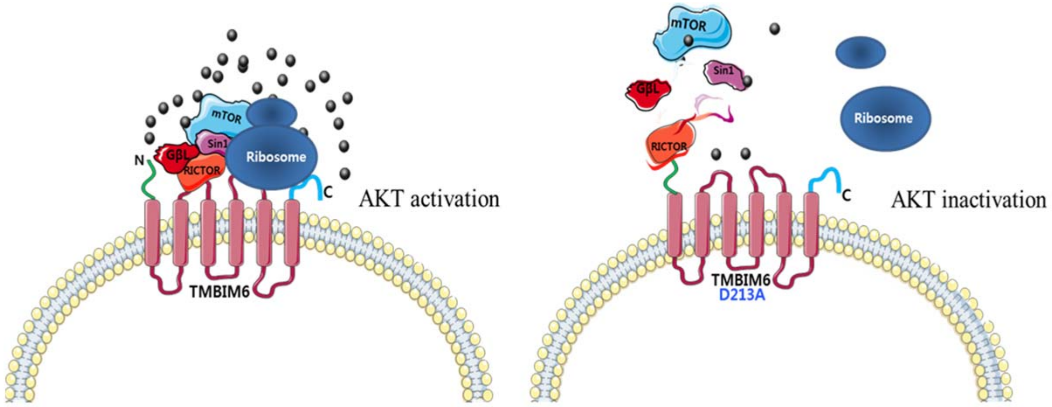

Supplementary Fig. 10 Ca^{2+} regulates mTORC2 activation. (A) PLA between the indicated proteins (red dots) in HT1080 cells treated with BAPTA-AM (10 μM), BAPTA (10 μM), and EGTA-AM (10 μM). Scale bar, 15 μm. Right; Right, quantification of red dots (n = 3 independent experiments). Data are presented as means ± SD. $^{**}p < 0.01$; $^{****}p < 0.0001$, one-way ANOVA followed by Tukey's post hoc test. (B) Illustration of TMBIM6-GCaMP3 by a genetically-encoded Ca^{2+} indicator (GCaMP3) fused directly to the C-terminus of TMBIM6 (TMBIM6-GCaMP3). (C) TMBIM6 and D213A expression-rescued KO cells were stained for calnexin (CANX, ER marker). (D) The scheme of TMBIM6 characteristics; TMBIM6-leaky Ca^{2+} and the interaction with mTORC2 and ribosome complex.

Supplementary Fig. 11

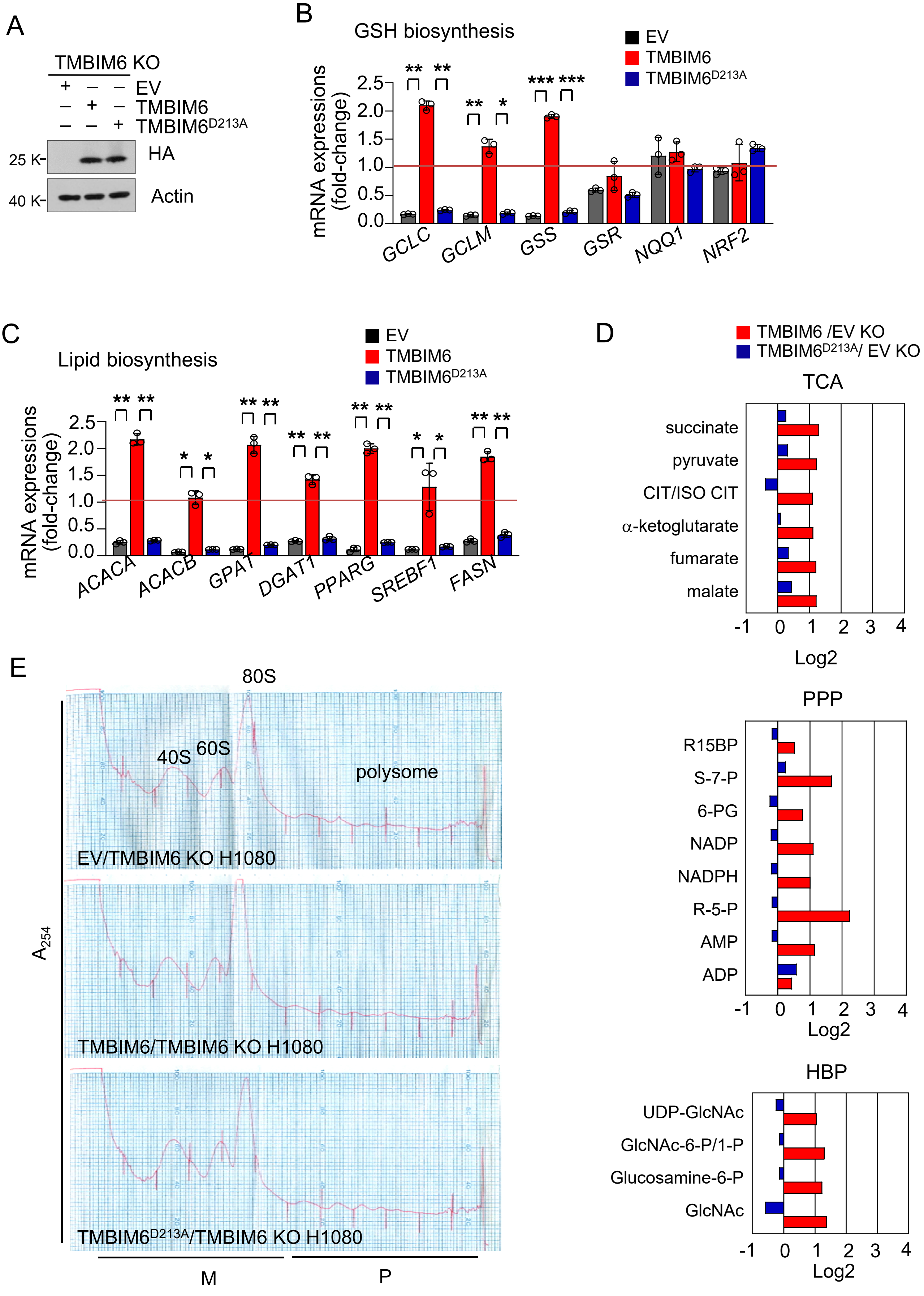

Supplementary Fig. 11 TMBIM6 regulates mTORC2-dependent metabolism. (A) Immunoblotting of TMBIM6-HA in empty vector, TMBIM6, TMBIM6 D213A-rescued TMBIM6 KO HT1080 cells. (B-C) mRNA levels of GSH biosynthesis genes (B) and *de novo* lipid biosynthesis genes (C) in empty vector, TMBIM6, TMBIM6 D213A-rescued TMBIM6 KO HT1080 cells, as determined by qRT-PCR. Quantification data represent the expression level of genes compared with those in normalized WT HT1080 cells (red line, n = 3 independent experiments). Data are presented as means ± SD. $^{*}p < 0.05$, $^{**}p < 0.01$, $^{***}p < 0.001$, two-way ANOVA followed by Bonferroni's post hoc test. (D) Metabolite analysis in empty vector, TMBIM6, TMBIM6 D213A-rescued TMBIM6 KO HT1080 cells. Quantification data represent the metabolite level compared with those in empty vector-rescued TMBIM6 KO HT1080 cells (n = 2 independent experiments). (E) Polysome profiling performed in empty vector, TMBIM6, TMBIM6 D213A-rescued TMBIM6 KO HT1080 cells by sucrose gradient fractionation.

Supplementary Fig. 12

A

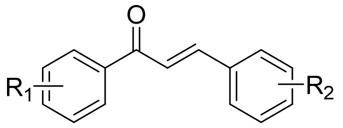

Chalcone scaffold

C

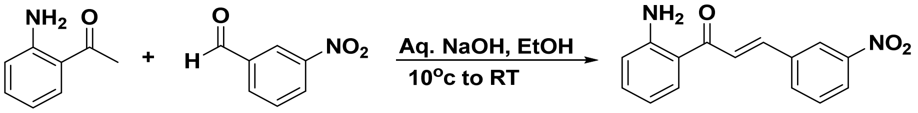

B

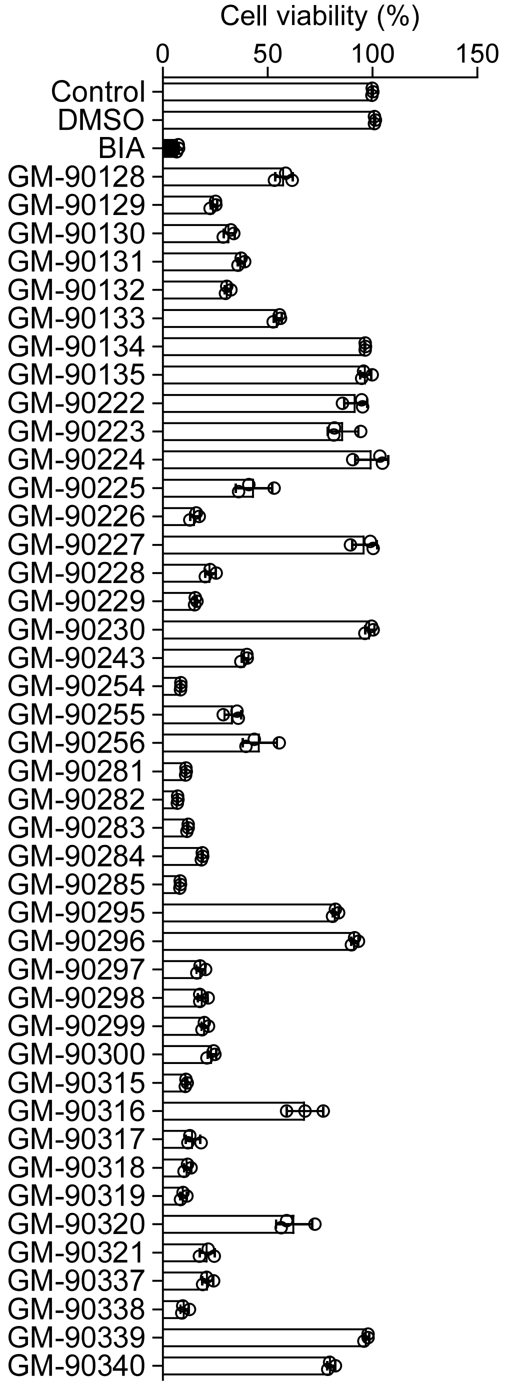

D

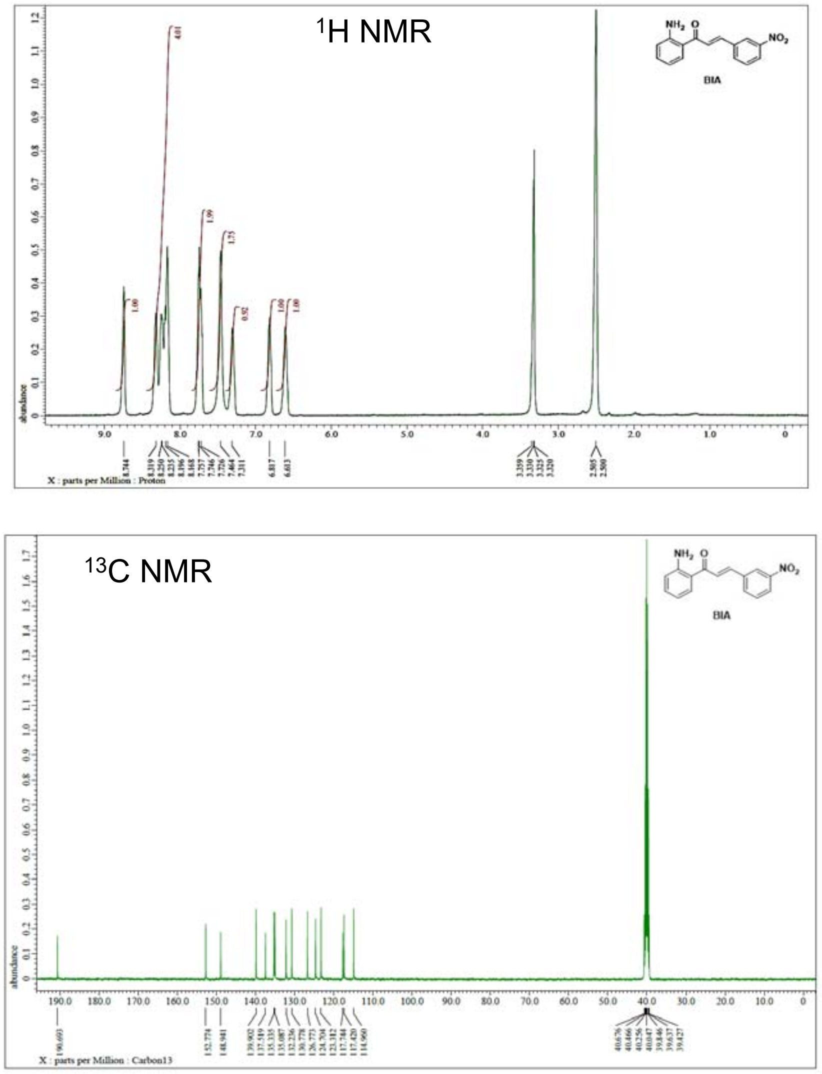

Supplementary Fig. 12 Synthesis of BIA. (A) Schematic representation of chalcone structure. (B) Cell viability of HT1080 cells with 10 uM of chalcone substituents (n = 3 independent experiments). Data are presented as means ± SD. One-way ANOVA followed by Tukey's post hoc test was used. (C) Schematic representation of the BIA synthesis. (D) NMR data of BIA.

Supplementary Fig. 13

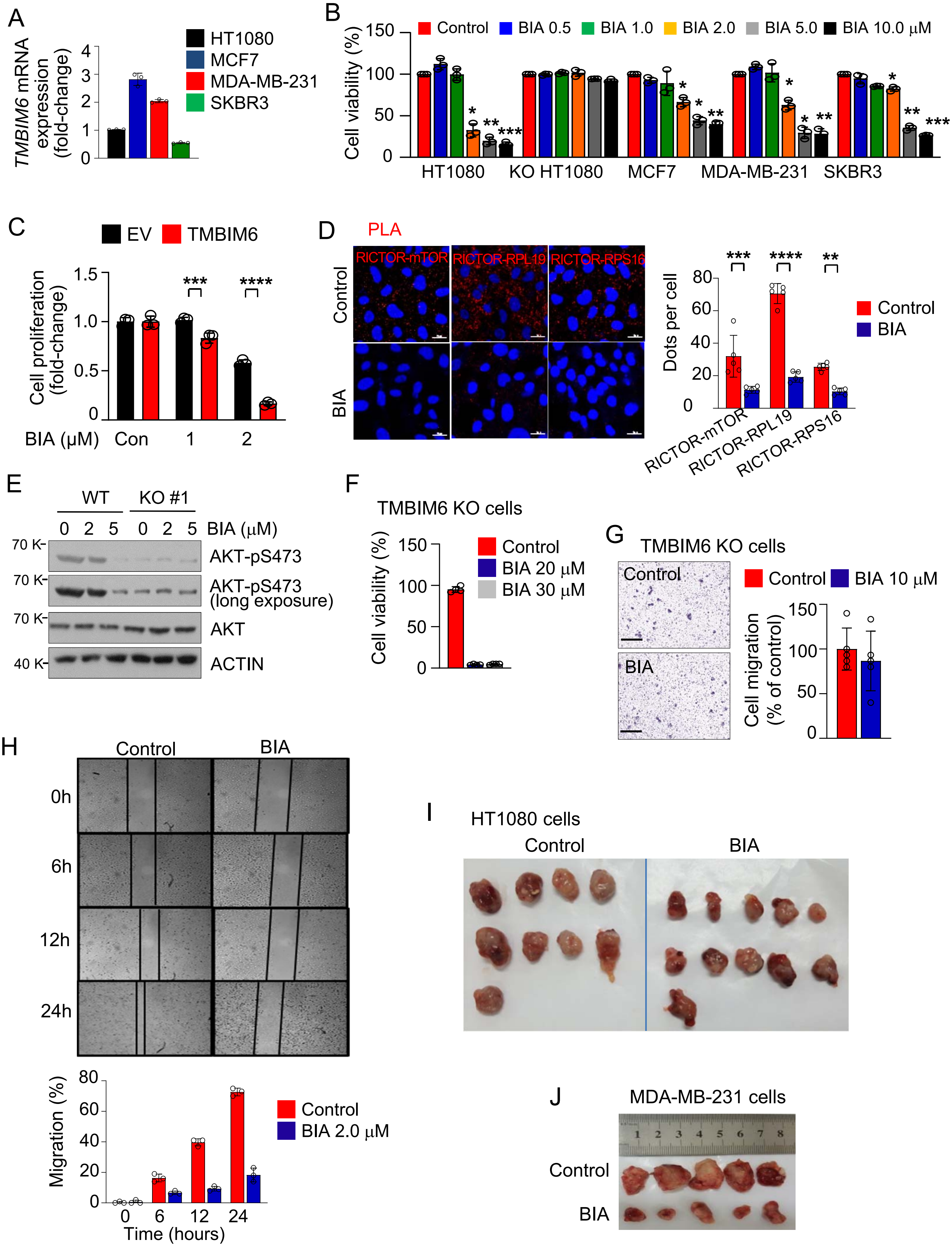

Supplementary Fig. 13 Inhibitory function of BIA in cancer progression. (A) TMBIM6 mRNA levels in various cancer cell lines. mRNA levels in each cells were normalized to the level of β-actin (n = 3 independent experiments). Data are presented as means ± SD. $*p < 0.05$; $**p < 0.01$; $***p < 0.001$, one-way ANOVA followed by Tukey's post hoc test. (B) Cell viability was measured in the indicated concentrations of BIA-treated cancer cells at three days (n = 3 independent experiments). (C) Proliferation of cells stably expressing TMBIM6 or the empty vector was analyzed after 1 day of treatment with indicated concentrations of BIA (n = 3 independent experiments). Data are presented as means ± SD. $***p < 0.001$, $****p < 0.0001$, two-way ANOVA followed by Bonferroni's post hoc test. (D) PLA between RICTOR and the following protein, mTOR, RPL19 and RPS 16 was performed in HT1080 cells with 10 μM BIA. Scale bar, 20 μm. Right, quantification of red dots (n = 5 independent experiments). Data are presented as means ± SD. $**p < 0.01$, $***p < 0.001$, $****p < 0.0001$, two-way ANOVA followed by Bonferroni's post hoc test. (E-G) Immunoblotting of phosphorylation of AKT (E), cell viability assay (F), and images of migrated cells and its quantification assay (G) were performed in TMBIM6 KO HT1080 cells treated with indicated concentrations of BIA. Quantification of cell viability and migrated cells in the BIA-treated cells normalized to control cells (n = 3 independent experiments). (H) The wound healing assay was performed with HT1080 cells treated with 2 μM BIA. Representative images (left) and quantification (right; n = 3 independent experiments) are shown (I and J). Representative images of *in vivo* tumors from xenograft experiments were shown in Figure 8 (n = 9 and 11 for control and treatment groups for HT1080 cells (I), and n = 6 mice per group for MDA-MB-231 cells (J)).

Supplementary Fig. 14

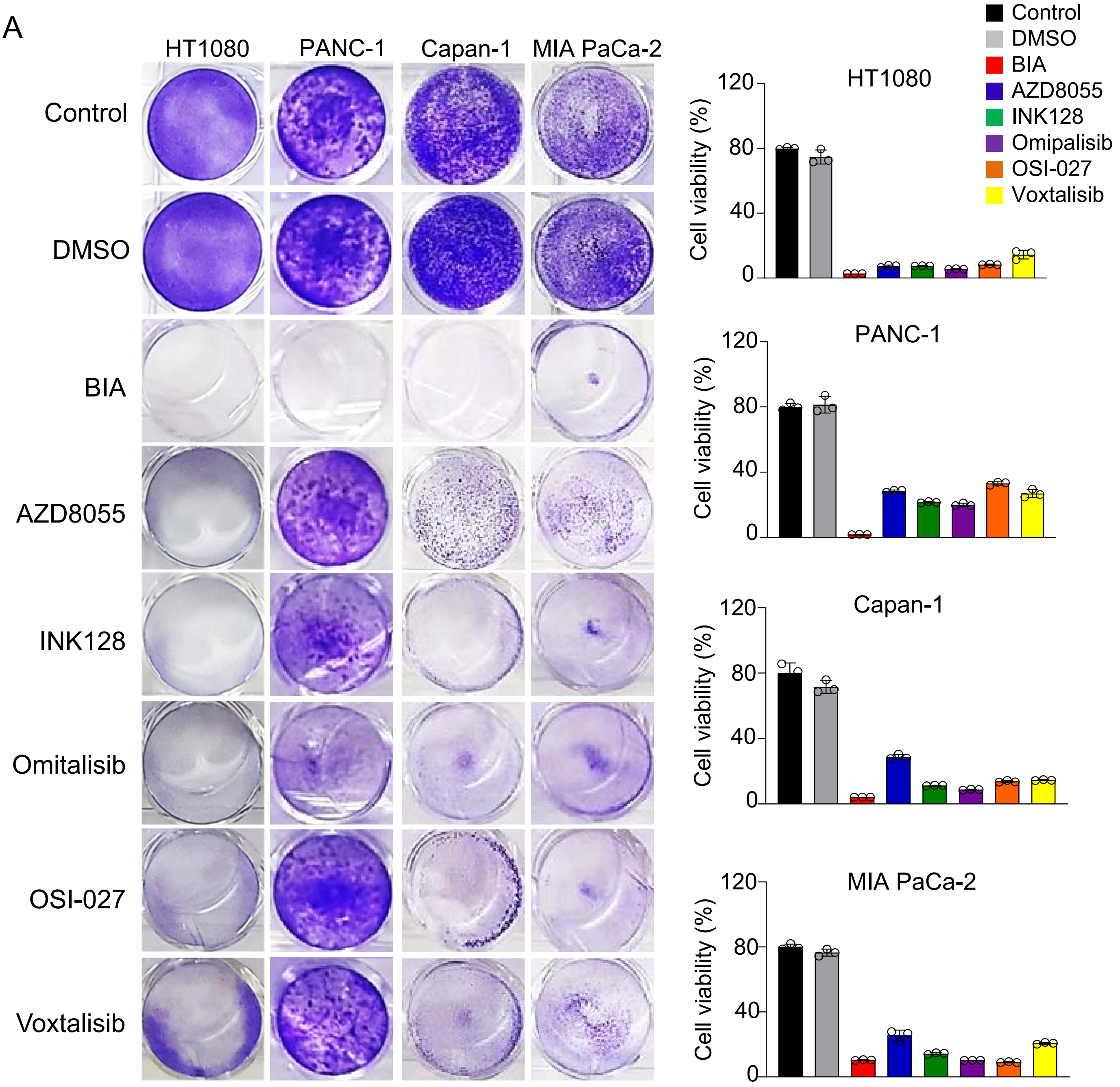

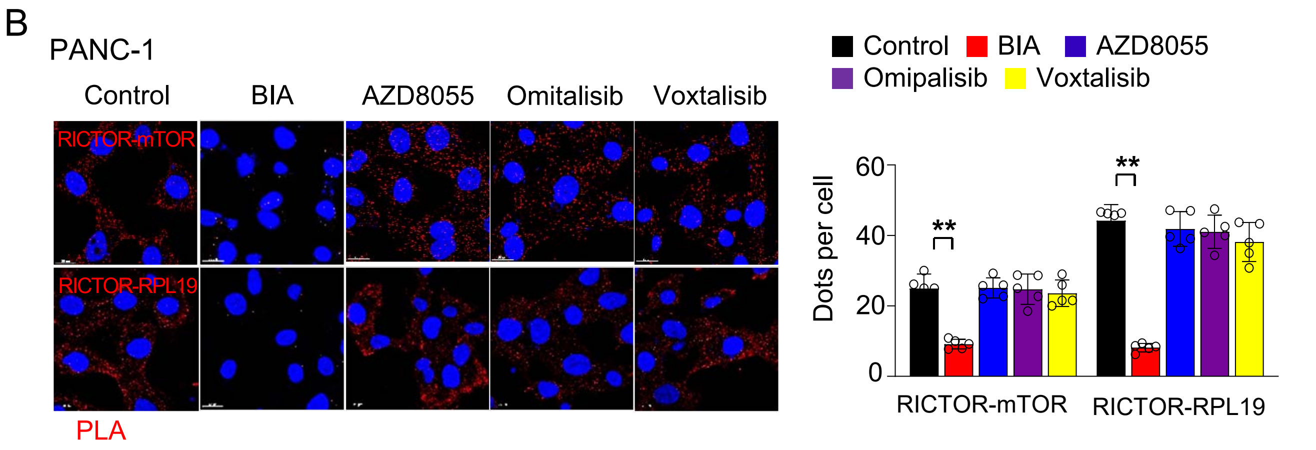

Supplementary Fig. 14 BIA decreases cell survival. (A) The images of crystal violet staining in HT1080, PANC-1, Capan-1, and MIA PaCa-2 cells after treatment with 10 μM BIA and mTOR inhibitors. Right; quantification of cell viability normalized to control cells (n = 3 independent experiments). (B) PLA between the indicated proteins (red dots) in BIA or mTOR inhibitors-treated PANC-1 cells. Right, quantification of red dots (n = 5 independent experiments). Scale bar, 20 μm. Data are presented as means ± SD. $^{**}p < 0.01$, two-way ANOVA followed by Bonferroni's post hoc test.

Supplementary Fig. 15 Uncropped images of blots (raw data)

Supplementary Fig. 15

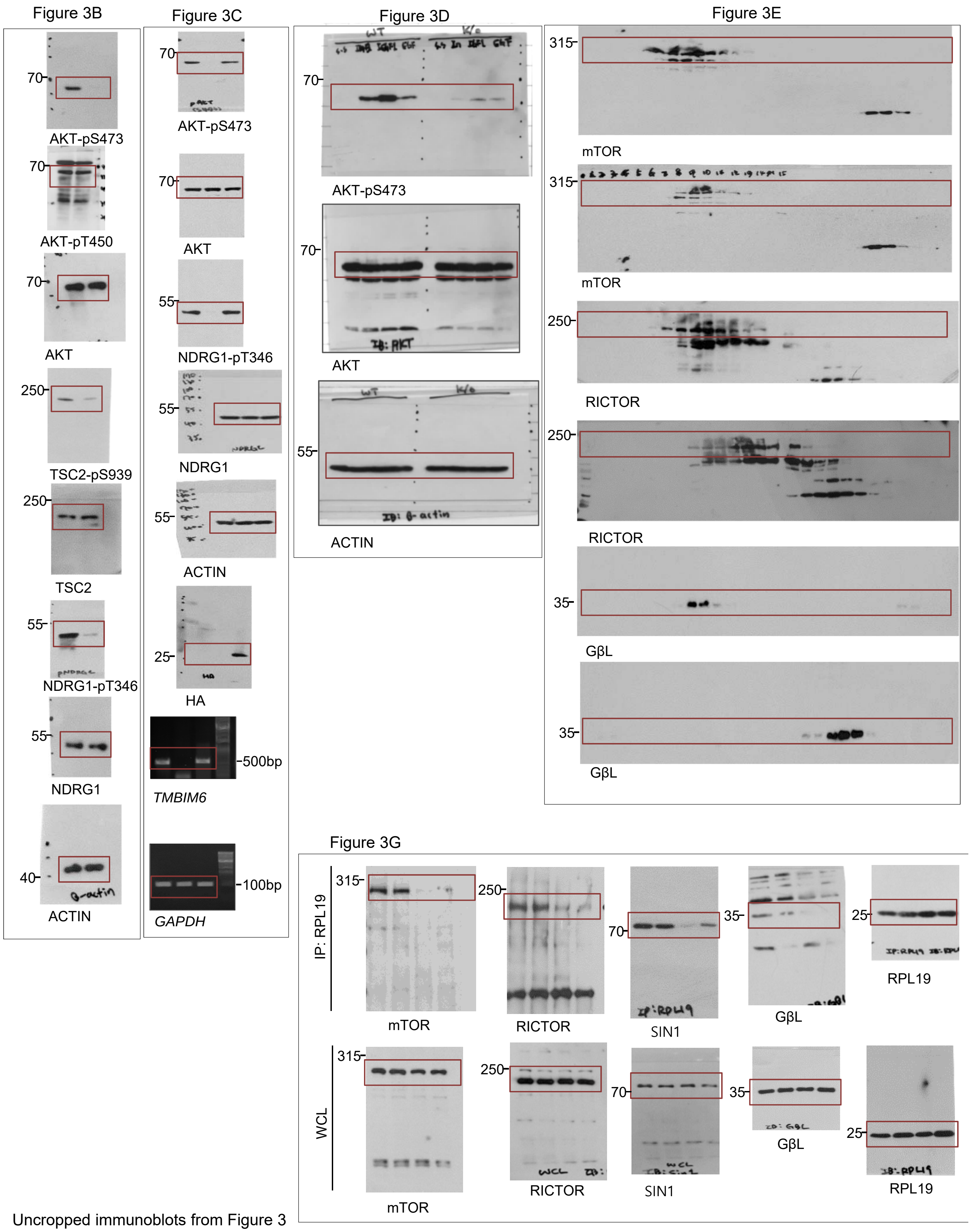

Uncropped immunoblots from Figure 3

Supplementary Fig. 15

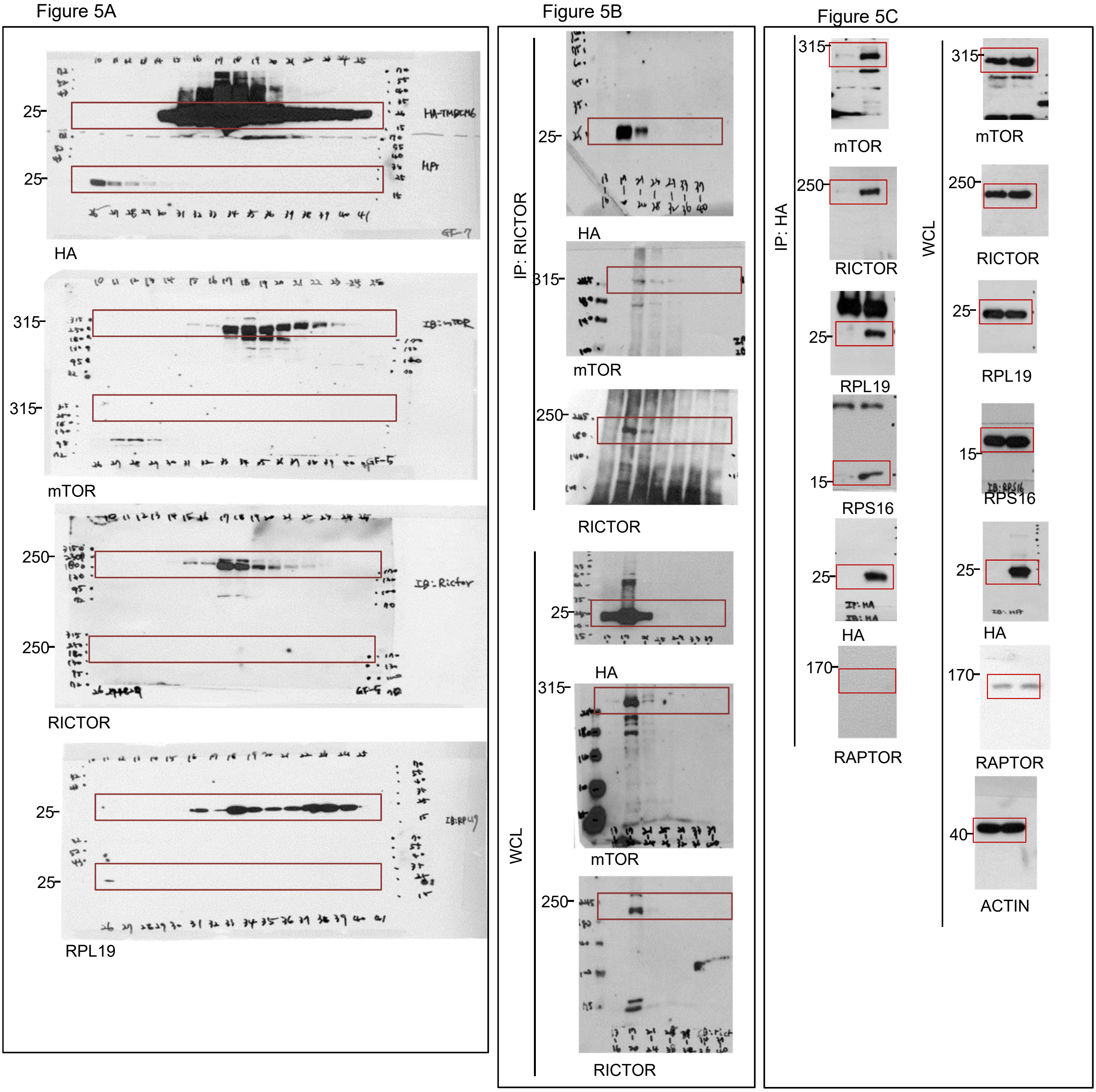

Uncropped immunoblots from Figure 5

Supplementary Fig. 15

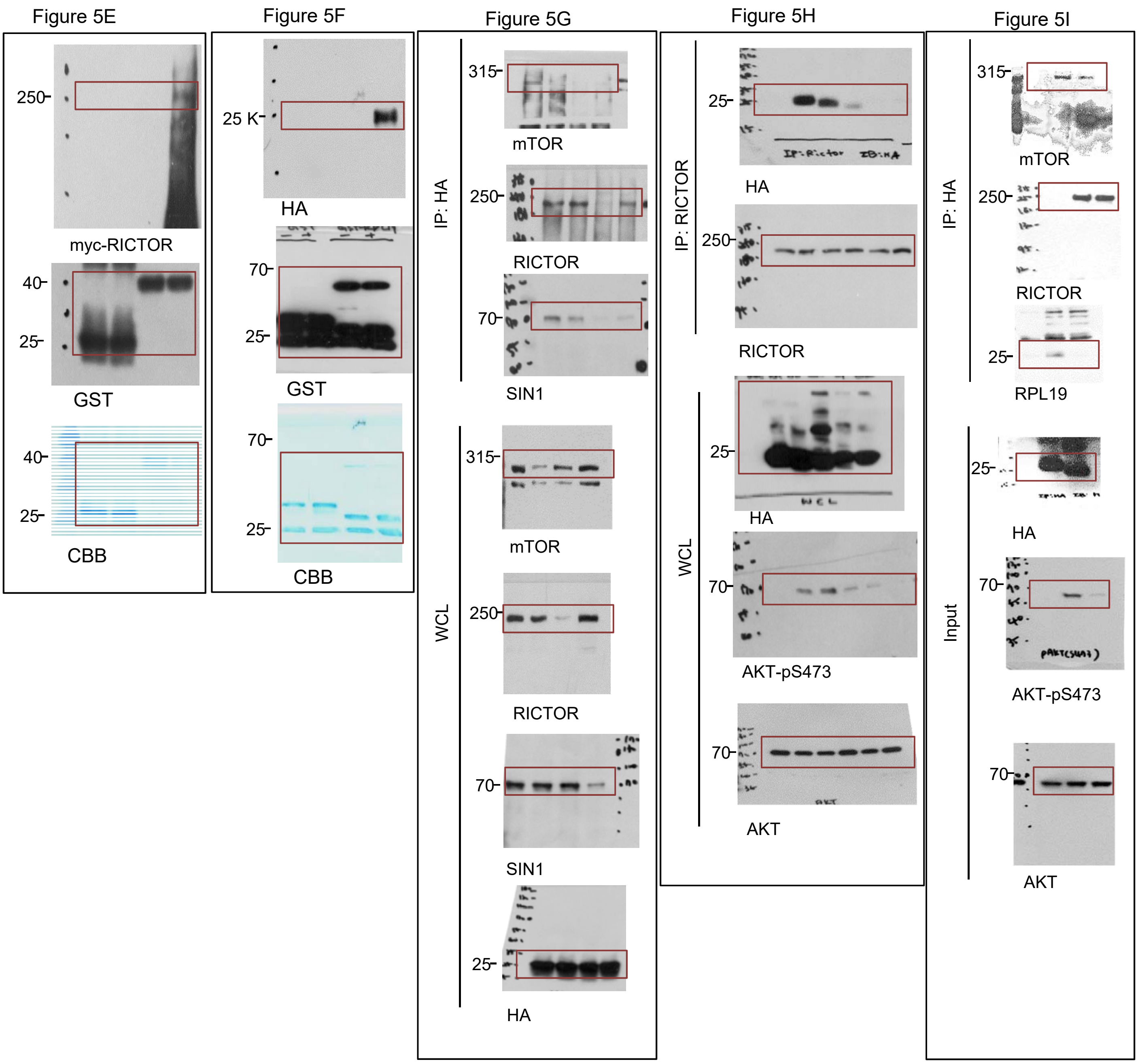

Uncropped immunoblots from Figure 5

Supplementary Fig. 15

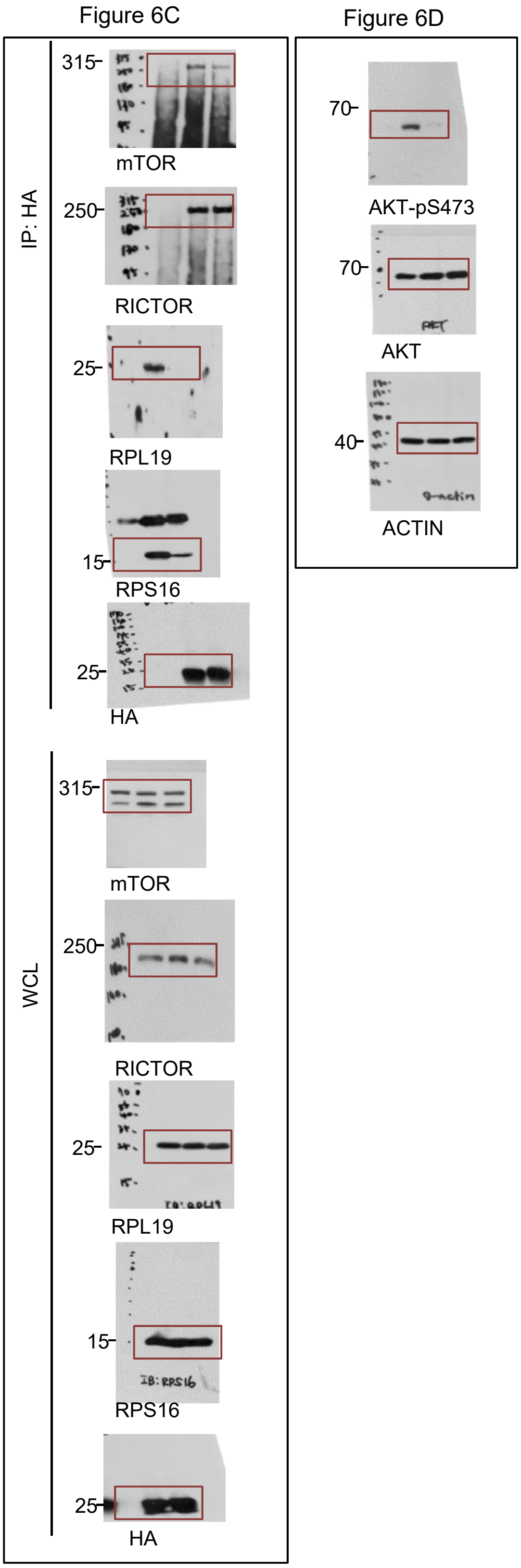

Uncropped immunoblots from Figure 6

Supplementary Fig. 15

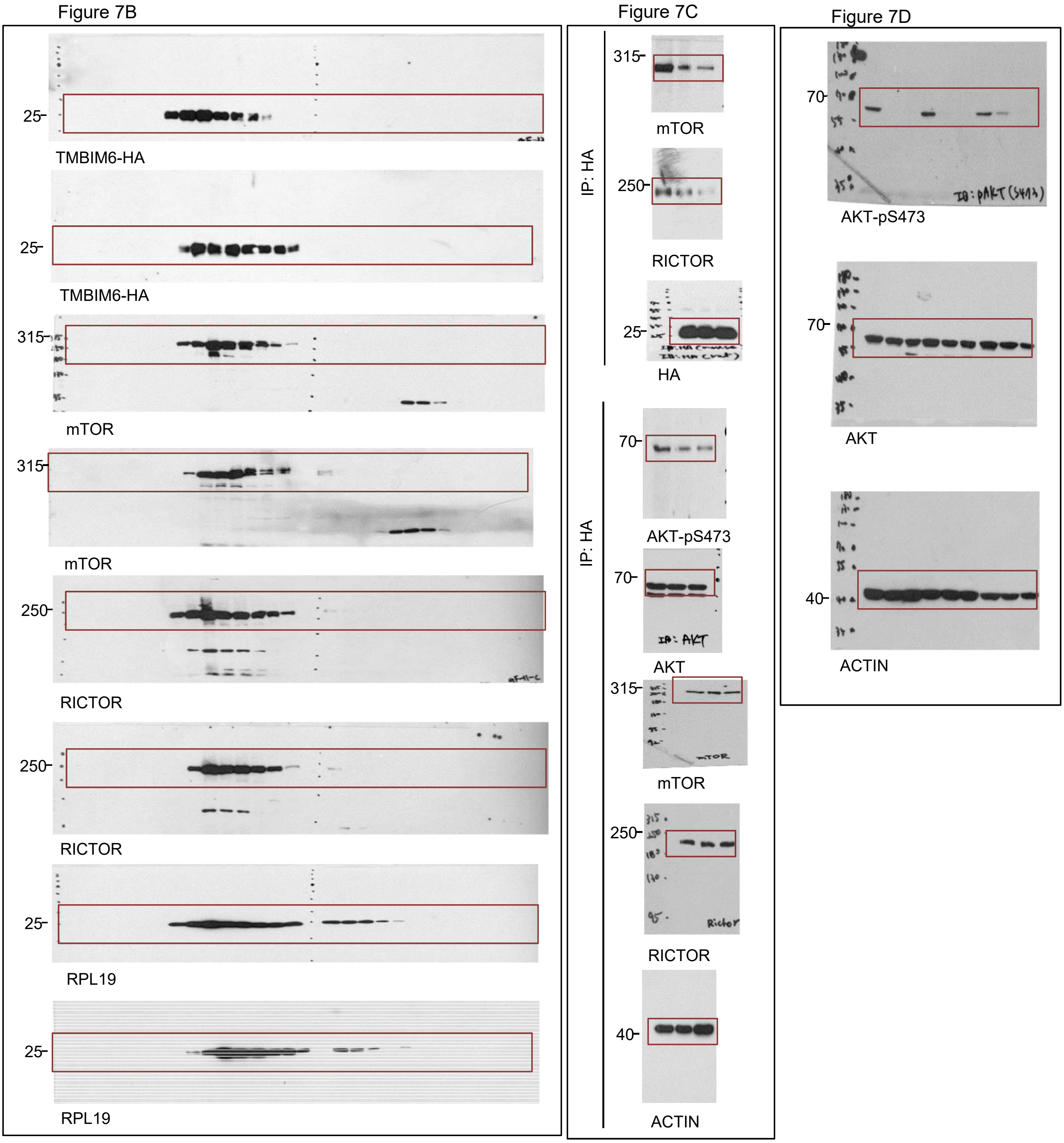

Uncropped immunoblots from Figure 7

Supplementary Fig. 15

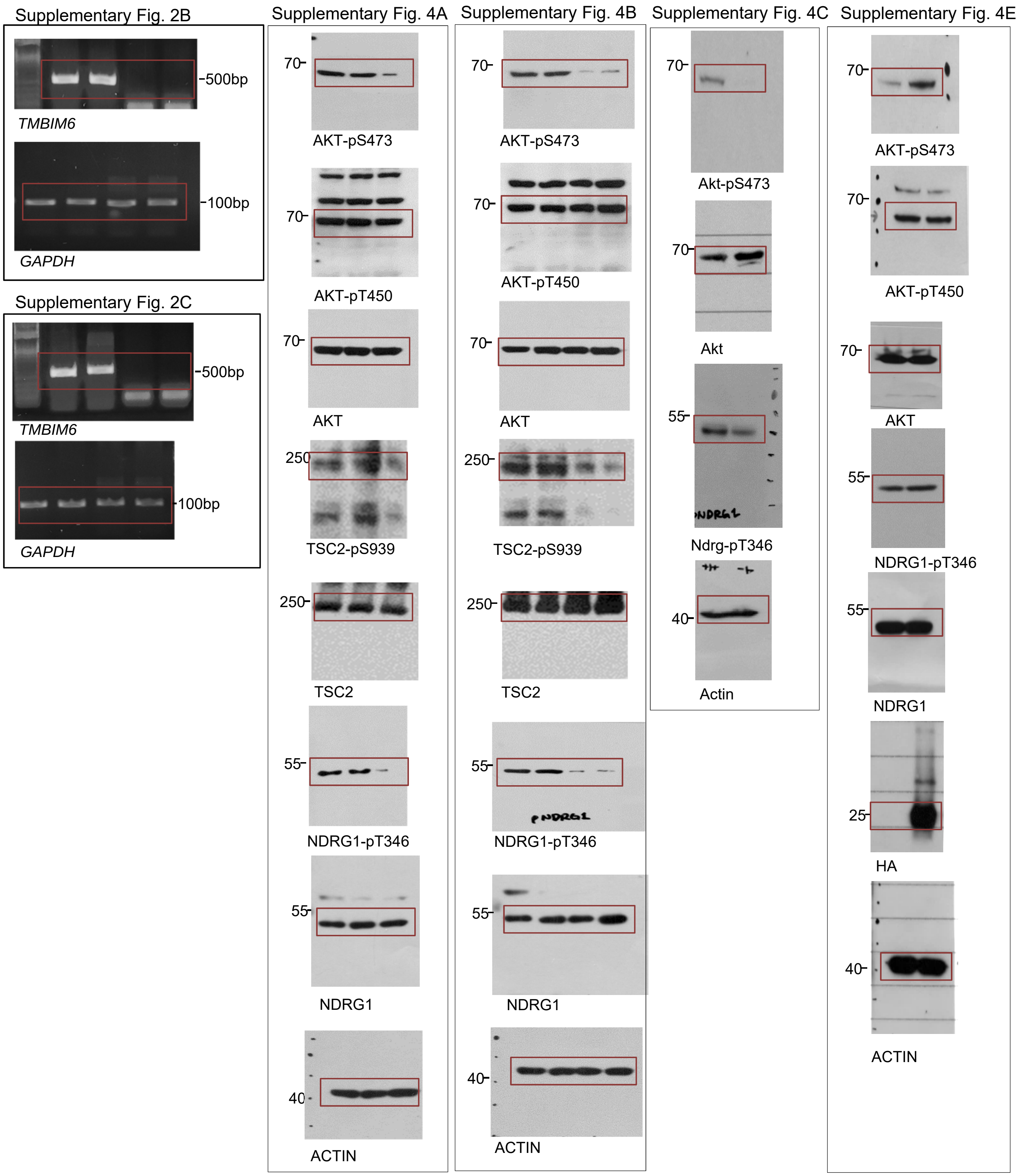

Uncropped immunoblots from Supplementary Figure 2 and 4

Supplementary Fig. 15

Supplementary Fig. 4F

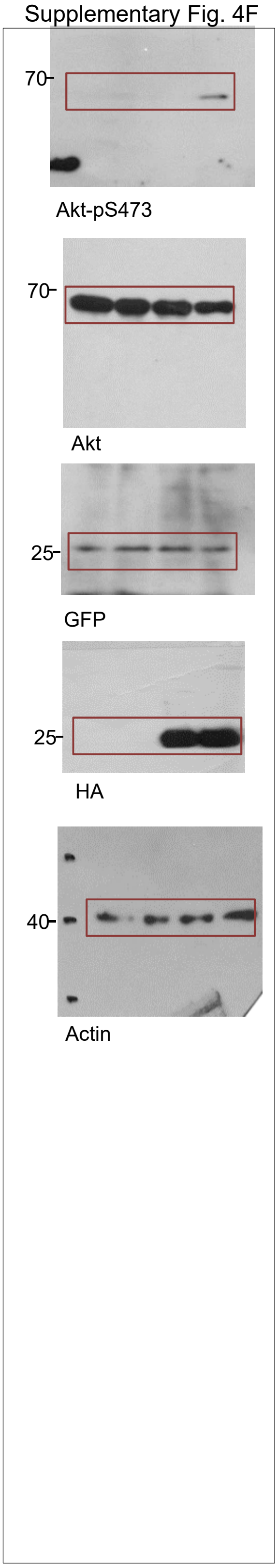

Supplementary Fig. 4G

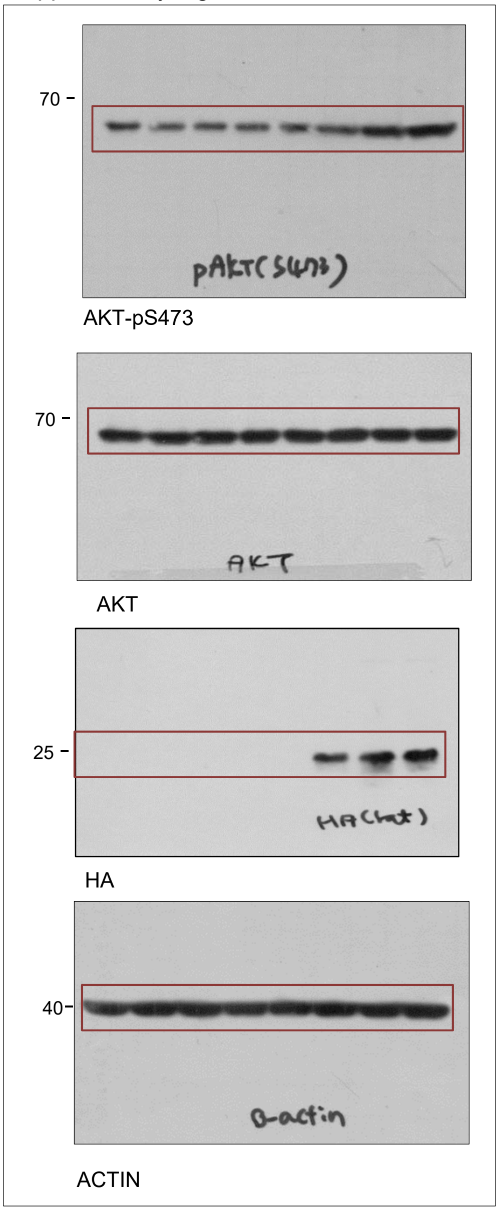

Supplementary Fig. 4J

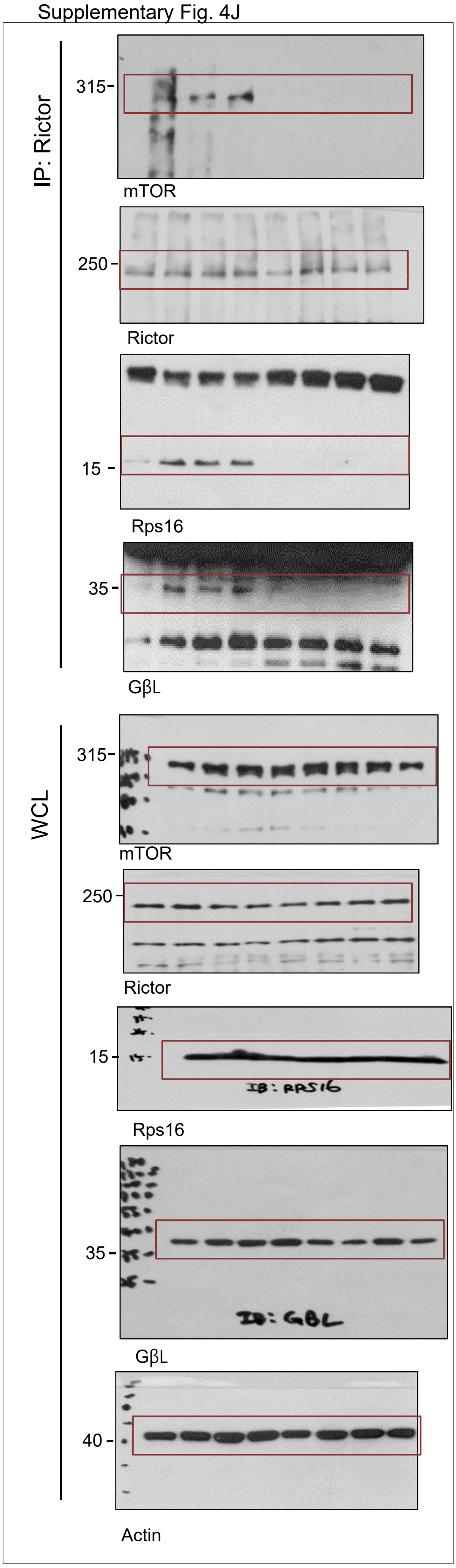

Uncropped immunoblots from Supplementary Figure 4

Supplementary Fig. 15

Supplementary Fig. 5B

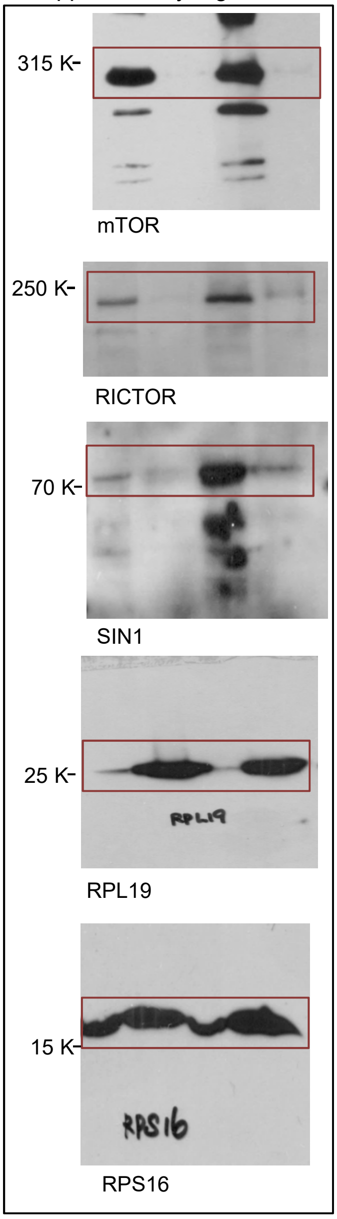

Supplementary Fig. 5C

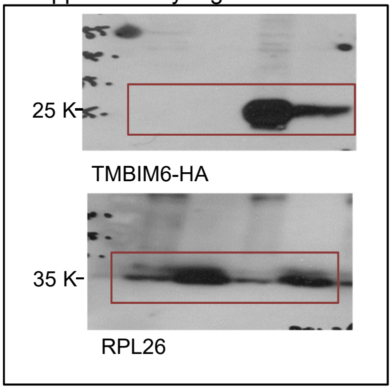

Supplementary Fig. 5D

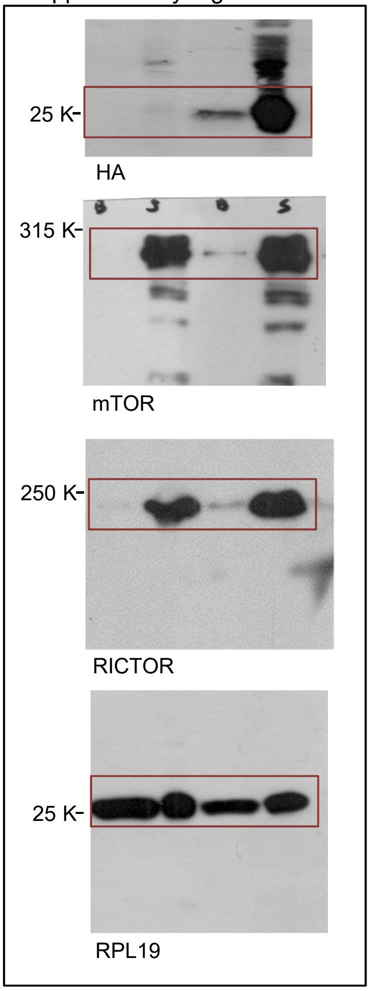

Supplementary Fig. 13E

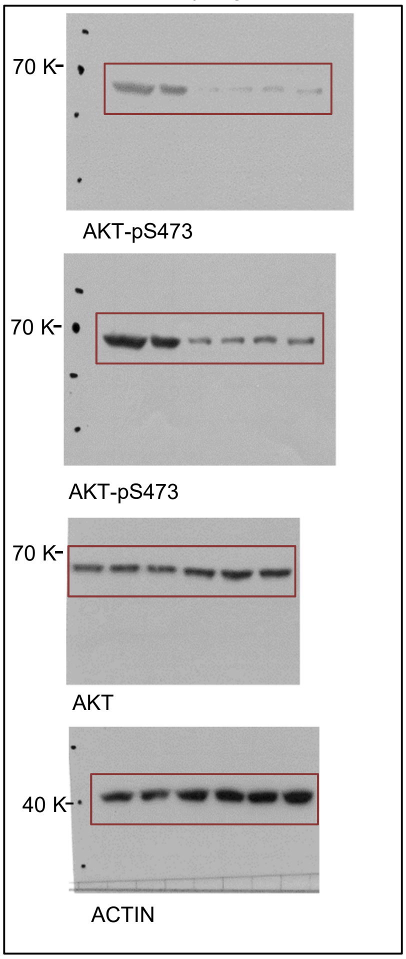

Supplementary Fig. 11A

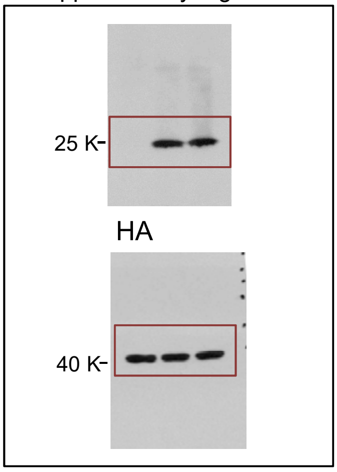

Uncropped immunoblots from Supplementary Figure 5, 11, and 13
