# Supplementary material for: TMBIM6/BI-1 contributes to cancer progression through assembly with mTORC2 and AKT activation
Source: Nat Commun. 2020 Aug 11;11:4012. doi: 10.1038/s41467-020-17802-4 (PMC7419509; doi:10.1038/s41467-020-17802-4)
Supplement: Supplementary file 3 — Description of Additional Supplementary Files [file 41467_2020_17802_MOESM3_ESM.pdf]

Description of Additional Supplementary Files

File Name: Supplementary Data 1
Description: Gene list from Microarray

File Name: Supplementary Data 2
Description: MS/MS data for RICTOR interacting proteins identified by LS/MS-MS analysis

File Name: Supplementary Data 3
Description: Primers for PCR amplification of human transcripts
