# Supplementary material for: TMBIM6/BI-1 contributes to cancer progression through assembly with mTORC2 and AKT activation
Source: Nat Commun. 2020 Aug 11;11:4012. doi: 10.1038/s41467-020-17802-4 (PMC7419509; doi:10.1038/s41467-020-17802-4)
Supplement: Supplementary file 7 — Reporting Summary [file 41467_2020_17802_MOESM7_ESM.pdf]

nature research

Corresponding author(s): Han-Jung Chae, Hyung-Ryong Kim

Last updated by author(s): 3rd July, 2020

Reporting Summary

Nature Research wishes to improve the reproducibility of the work that we publish. This form provides structure for consistency and transparency in reporting. For further information on Nature Research policies, see our Editorial Policies and the Editorial Policy Checklist.

Statistics

For all statistical analyses, confirm that the following items are present in the figure legend, table legend, main text, or Methods section.

n/a	Confirmed	
☐	☒	The exact sample size (*n*) for each experimental group/condition, given as a discrete number and unit of measurement
☐	☒	A statement on whether measurements were taken from distinct samples or whether the same sample was measured repeatedly
☐	☒	The statistical test(s) used AND whether they are one- or two-sided<br>*Only common tests should be described solely by name; describe more complex techniques in the Methods section.*
☒	☐	A description of all covariates tested
☐	☒	A description of any assumptions or corrections, such as tests of normality and adjustment for multiple comparisons
☐	☒	A full description of the statistical parameters including central tendency (e.g. means) or other basic estimates (e.g. regression coefficient) AND variation (e.g. standard deviation) or associated estimates of uncertainty (e.g. confidence intervals)
☐	☒	For null hypothesis testing, the test statistic (e.g. *F*, *t*, *r*) with confidence intervals, effect sizes, degrees of freedom and *P* value noted<br>*Give P values as exact values whenever suitable.*
☒	☐	For Bayesian analysis, information on the choice of priors and Markov chain Monte Carlo settings
☒	☐	For hierarchical and complex designs, identification of the appropriate level for tests and full reporting of outcomes
☐	☒	Estimates of effect sizes (e.g. Cohen's *d*, Pearson's *r*), indicating how they were calculated

Our web collection on statistics for biologists contains articles on many of the points above.

Software and code

Policy information about availability of computer code

Data collection	Microarray data : OpArray Human Genome 35K array (OPHSV4; Operon Biotechnologies, GmbH), GeneChip Human Gene 2.0 ST oligonucleotide arrays (Affymetrix, Santa Clara, CA, USA).<br>qRT-PCR data : ABI PRISM 7700 Sequence Detection System (Applied Biosystems)
Data analysis	Statistical analysis : GraphPad Prism v.8 software<br>Microarray data analysis : GenePix Pro v.5.1 (Axon Instruments) and GeneSpring GX v.7.3.1 (Silicon Genetics, Redwood City, CA, USA), Expression Console v.1.1 software (Affymetrix)<br>Publicly available Gene Expression Omnibus (GEO) datasets : GEO2R<br>Overall survival analysis of cancer patient samples in TCGA datasets : OncoLnc (http://www.oncolnc.org, No version), GEPIA2 (http://gepia2.cancer-pku.cn)<br>Western Blot quantitation : ImageJ 1.52k<br>Image : AxioVision v.4.3 software (Carl Zeiss, Oberkochen, Germany)<br>Live image : ZEN 2.5 Lite

For manuscripts utilizing custom algorithms or software that are central to the research but not yet described in published literature, software must be made available to editors and reviewers. We strongly encourage code deposition in a community repository (e.g. GitHub). See the Nature Research guidelines for submitting code & software for further information.

Data

Policy information about availability of data

All manuscripts must include a data availability statement. This statement should provide the following information, where applicable:

- Accession codes, unique identifiers, or web links for publicly available datasets
- A list of figures that have associated raw data
- A description of any restrictions on data availability

Data availability
All data generated or analysed in this study are included in the published article and Supplementary Information file are available from the corresponding authors upon reasonable request. All raw data used for generating figures are provided as a Source Data file. The microarray data have been deposited to GEO under accession number GSE153716.

Field-specific reporting

Please select the one below that is the best fit for your research. If you are not sure, read the appropriate sections before making your selection.

☒ Life sciences ☐ Behavioural & social sciences ☐ Ecological, evolutionary & environmental sciences

For a reference copy of the document with all sections, see nature.com/documents/nr-reporting-summary-flat.pdf

Life sciences study design

All studies must disclose on these points even when the disclosure is negative.

Sample size	No statistical method was used to pre-determine the sample size, but the sample sizes were chosen based on previous publications , which were sufficient for statistical analysis.
Data exclusions	No data were excluded.
Replication	The experimental results were reproduced, and the replication numbers are described in the corresponding figure legends.
Randomization	Animals were randomly allocated for each group as described in Methods under Animal Studies subsection.
Blinding	Histology including IHC, confocal microscopy data were performed blindly and analysis was done in a non-blind fashion. Data collection of most mouse tumor experiments were performed in a blinding manner. Other cellular work were not performed in a blinding manner.

Reporting for specific materials, systems and methods

We require information from authors about some types of materials, experimental systems and methods used in many studies. Here, indicate whether each material, system or method listed is relevant to your study. If you are not sure if a list item applies to your research, read the appropriate section before selecting a response.

Materials & experimental systems

n/a	Involved in the study	
☐	☒	Antibodies
☐	☒	Eukaryotic cell lines
☒	☐	Palaeontology and archaeology
☐	☒	Animals and other organisms
☒	☐	Human research participants
☒	☐	Clinical data
☒	☐	Dual use research of concern

Methods

n/a	Involved in the study	
☒	☐	ChIP-seq
☒	☐	Flow cytometry
☒	☐	MRI-based neuroimaging

Antibodies

Antibodies used	Antibodies against the following proteins were used in this study: TMBIM6/BI-1 (1:100, ab51905), and RPL19 (1:1000, ab128648) (all from Abcam, Cambridge, UK); RICTOR (1:2000, A300-459A) (Bethyl Laboratories, Montgomery, TX, USA); AKT (1:1000, #9272), GST (1:1000, #2622), mTOR (1:1000, #2972), mTOR (1:1000, #4517), NDRG1 (1:1000, #9408), phospho-Ser473-AKT (1:1000, #9271), phospho-Ser939-TSC2 (1:1000, #3615), phospho-Thr308-AKT (1:1000, #4056), TSC2 (1:1000, #4308), phospho-Thr346-NDRG1 (1:1000, #3217), SIN1 (1:1000, #12860), p70 S6 Kinase (1:100, #9202), RICTOR (1:1000, #2114), RICTOR (Sepharose bead conjugate, #5379), and RAPTOR (1:1000, #2280) (all from Cell Signaling Technology, Danvers, MA, USA); HA (1:2000, 11867423001) (Roche Diagnostics, Basel, Switzerland); actin (1:1000, sc-47778), Ki-67 (1:100, sc-15402), phospho-Thr450-AKT (1:1000, sc-293094), RPL19 (1:1000, sc-100830), and RPS16 (1:1000, sc-102087), (all from Santa Cruz Biotechnology, Santa Cruz, CA, USA). Secondary antibodies

	(1:10,000) for immunoblotting (Jackson ImmunoResearch, West Grove, PA, USA) and immunoprecipitation (1:5000, sc-2006) (Santa Cruz Biotechnology) were also used.
Validation	All primary antibodies used in this study were validated by the manufacturer company. Validation data / citations can be found on the manufacture website by searching the antibody catalog number provided in materials and methods section of our manuscript.

Eukaryotic cell lines

Policy information about cell lines

Cell line source(s)	HeLa, HT1080, MCF7, MDA-MB-231, SKBR3, PANC-1, Capan-1, and MIA PaCa-2 were purchased from the Korean Cell Line Bank (Seoul, Korea). MEF, TMBIM6 KO HT1080 cell lines were generated in the lab. T-REx-293 cell line was purchased from Thermo Fisher Scientific.
Authentication	The cell lines were not authenticated.
Mycoplasma contamination	All cell lines were routinely tested for mycoplasma using the e-Myco™ Mycoplasma PCR Detection Kit (iNtRON biotechnology; 25235). All the cells lines were tested negative for mycoplasma contamination.
Commonly misidentified lines (See ICLAC register)	None

Animals and other organisms

Policy information about studies involving animals; ARRIVE guidelines recommended for reporting animal research

Laboratory animals	All animals were maintained in the Chonbuk National University specific pathogen free (SPF) facility. Six- to eight-week female BklNbt:BALB/c/nu/nu mice (Damul, Daejeon, Korea) were used for tumor xenografts. Mice were housed (n = 5/cage) in a fully climate-controlled room at constant temperature and humidity on a 12:12-h light/dark cycle with free access to food and water. 48 and 120 hours post-fertilization (hpf) of Zebrafish embryos were used. Zebrafish experiments were conducted according to the ethics in animal experiments. However, approval for the study is exempted for Zebrafish experiments where less than 120 hpf are used.
Wild animals	N/A
Field-collected samples	N/A
Ethics oversight	Animal experiments were performed in accordance with the Guide for the Care and Use of Laboratory Animals of Chonbuk National University Institutional Animal Care and Use Committee (Jeonju, Korea; approval nos. CBNU 2015-064, CBNU 2016-56, and CBNU 2017-0026, and CBNU 2020-033) and related ethics regulations of the university.

Note that full information on the approval of the study protocol must also be provided in the manuscript.
